# The Rap activator Gef26 regulates synaptic growth and neuronal survival via inhibition of BMP signaling

**DOI:** 10.1186/s13041-017-0342-7

**Published:** 2017-12-28

**Authors:** Keunjung Heo, Minyeop Nahm, Min-Jung Lee, Young-Eun Kim, Chang-Seok Ki, Seung Hyun Kim, Seungbok Lee

**Affiliations:** 1 Department of Brain and Cognitive Sciences, College of Natural Sciences, Seoul National University, Seoul, 08826 South Korea; 2 Department of Cell & Developmental Biology, Dental Research Institute, Seoul National University, Seoul, 03080 South Korea; 3 Department of Neurology, Hanyang University College of Medicine, Seoul, 04763 South Korea; 4 Department of Laboratory Medicine and Genetics, Samsung Medical Center, Sungkyunkwan University School of Medicine, Seoul, 06351 South Korea

**Keywords:** *Drosophila*, Gef26, Synaptic growth, Neurodegeneration, Endocytic regulation of BMP signaling

## Abstract

In *Drosophila*, precise regulation of BMP signaling is essential for normal synaptic growth at the larval neuromuscular junction (NMJ) and neuronal survival in the adult brain. However, the molecular mechanisms underlying fine-tuning of BMP signaling in neurons remain poorly understood. We show that loss of the *Drosophila* PDZ guanine nucleotide exchange factor Gef26 significantly increases synaptic growth at the NMJ and enhances BMP signaling in motor neurons. We further show that Gef26 functions upstream of Rap1 in motor neurons to restrain synaptic growth. Synaptic overgrowth in *gef26* or *rap1* mutants requires BMP signaling, indicating that Gef26 and Rap1 regulate synaptic growth via inhibition of BMP signaling. We also show that Gef26 is involved in the endocytic downregulation of surface expression of the BMP receptors thickveins (Tkv) and wishful thinking (Wit). Finally, we demonstrate that loss of Gef26 also induces progressive brain neurodegeneration through Rap1- and BMP signaling-dependent mechanisms. Taken together, these results suggest that the Gef26-Rap1 signaling pathway regulates both synaptic growth and neuronal survival by controlling BMP signaling.

**Electronic supplementary material:**

The online version of this article (10.1186/s13041-017-0342-7) contains supplementary material, which is available to authorized users.

## Introduction

Transsynaptic retrograde signaling from postsynaptic cells controls the development and survival of presynaptic neurons [1–3]. At the *Drosophila* larval neuromuscular junction (NMJ), the bone morphogenetic protein (BMP) ligand glass bottom boat (Gbb) is secreted from the postsynaptic muscle and acts as a key retrograde signal that promotes the expansion of synaptic arbors [4–7]. In motoneurons, the Gbb signal is processed by a tetrameric presynaptic complex containing the type II BMP receptor wishful thinking (Wit) and either of two type II BMP receptors, thickveins (Tkv) and saxophone (Sax). Upon Gbb binding, this receptor complex phosphorylates the R-Smad mothers against decapentaplegic (Mad). Phosphorylated Mad (P-Mad) translocates into the nucleus through its interaction with the co-Smad Medea to regulate transcription of target genes [8]. Mutations disrupting this canonical BMP signaling pathway, including *gbb*, *wit*, *tkv*, *sax*, and *mad*, all display NMJ undergrowth and defective basal transmission [4–7]. In sharp contrast, genetic conditions to elevate presynaptic BMP signaling cause NMJ overgrowth with excessive formation of small “satellite” boutons [9–12], which bud off the main axis of the motor axon terminal. Based on these findings, it has been proposed that the level of BMP signaling is instructive for the regulation of NMJ synapse growth [10]. Subsequent work on the *Drosophila* brain has begun to reveal the importance of precise regulation of BMP signaling in the maintenance of adult neurons. It has been demonstrated that, in addition to synaptic overgrowth, elevation of BMP signaling induces abnormal brain neurodegeneration in the adult fly [9].

*Drosophila* NMJ studies have identified various endocytic proteins as negative regulators of BMP-dependent synaptic growth. For example, loss of two endocytosis regulators, Dap160/intersectin and endophilin, leads to an increase in synaptic P-Mad levels and NMJ overgrowth with excessive satellite bouton formation [10, 13]. In addition, a similar phenotype is also induced by loss of spichthyin (Spict), Spartin, and endosomal maturation defective (Ema), all of which are involved in endolysosomal trafficking of BMP receptors [9, 11, 14]. Importantly, these endocytic genes are shown to functionally interact with BMP signaling pathway components at the NMJ [9–11, 14]. These findings imply that endocytosis and subsequent lysosomal degradation of BMP receptors are important mechanisms involved in attenuating Gbb-induced signaling at the NMJ.

In a genetic screen for mutations that affect synaptic morphology at the *Drosophila* NMJ, we identified the *gef26* gene, which encodes a PDZ guanine nucleotide exchange factor (PDZ-GEF) for the small GTPase Rap1. Gef26 was originally known to control the development of various organs primarily by regulating cadherin-mediated cell-cell adhesion and integrin-dependent cell-matrix interactions [15–19]. Here, we report a novel role for the Gef26-Rap1 pathway in the regulation of BMP-dependent synaptic growth and neuronal survival. Null mutations in the *gef26* or *rap1* gene cause NMJ overgrowth characterized by excessive satellite bouton formation, recapitulating the phenotype induced by elevated BMP signaling. Genetic interactions between *gef26*, *rap1*, and components of the BMP pathway suggest that Gef26 acts through Rap1 to restrain BMP-dependent synaptic growth at the NMJ. Importantly, Gef26 promotes endocytic downregulation of surface expression of the BMP receptors Tkv and Wit. Finally, our genetic data indicate that regulation of BMP signaling by the Gef26-Rap1 pathway is critical for neuronal survival in the adult brain.

## Results

### *Drosophila gef26* is required presynaptically for normal synaptic growth

To identify genes involved in the regulation of synaptic development, we performed an anatomical screen on 1500 independent EP insertion lines [20, 21]. We inspected third instar larval NMJs using the axonal membrane marker anti-HRP. In this screen, we isolated an insertion (G3533) localized in the first intron of the *Drosophila gef26* gene (CG9491). These mutants displayed NMJ overgrowth with an excessive formation of small “satellite” boutons (data not shown), which protrude from parental boutons located at primary axon terminal arbors.

**Fig. 1:**
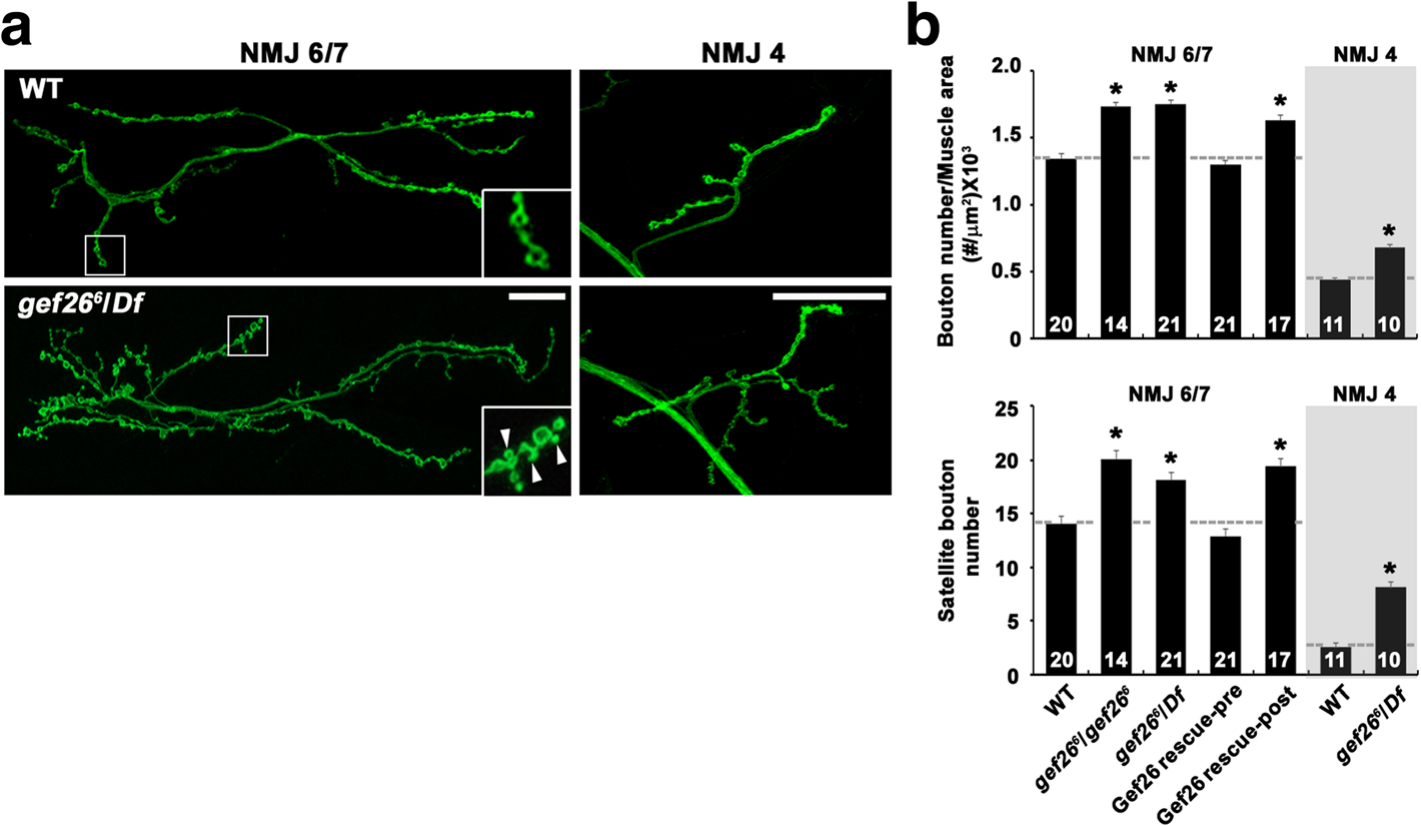
Loss of presynaptic *gef26* function leads to synaptic overgrowth at the NMJ. **a** Confocal images of anti-HRP-labeled NMJs 6/7 (left) and NMJs 4 (right) in wild-type (*w* ^*1118*^) and *gef26* ^*6*^/*Df* third-instar larvae. *gef26* mutant NMJs have increased numbers of total and satellite boutons compared with wild type. Arrowheads mark satellite boutons. Scale bars, 20 μm. **b** Quantification of total bouton number normalized to muscle surface area and satellite bouton number at NMJ 6/7 and NMJ 4 in wild-type, *gef26* ^*6*^/*gef26* ^*6*^, *gef26* ^*6*^/*Df*, *C155-GAL4*/+; *gef26* ^*6*^/*Df*; *UAS-gef26*/+ (Gef26 rescue-pre), and *gef26* ^*6*^/*Df*; *BG57-GAL4*/*UAS-gef26* (Gef26 rescue-post) third-instar larvae. The number of NMJs analyzed is indicated in each bar. Data are expressed as mean ± SEM. All comparisons are made with wild-type (*P < 0.001)

To determine the null phenotype of *gef26* at the NMJ, we utilized the transheterozygous combination of *gef26* ^*6*^, a previously reported null allele [19, 22], and the *Df(2 L)BSC5* deficiency (henceforth referred to as *Df*) to delete the *gef26* locus. A significant synaptic overgrowth phenotype was observed at every glutamatergic type-I NMJ in *gef26* ^*6*^ */Df* third instar larvae. To quantify the *gef26* phenotype, we measured overall bouton number and satellite bouton number at NMJ 6/7 and NMJ 4 from abdominal segment 2 (Fig. 1a, b; Additional file 1: Table S1). Compared with wild-type controls (*w* ^*1118*^), bouton number normalized to muscle surface area in *gef26* ^*6*^ */Df* larvae was increased by 24% at NMJ 6/7 and by 51% at NMJ 4. At the same time, satellite bouton number in *gef26* ^*6*^ */Df* was increased by 39% at NMJ 6/7 and by 219% at NMJ 4. Comparable synaptic growth defects were observed in larvae homozygous for *gef26* ^*6*^ (Fig. 1a, b).

To determine whether *gef26* function is required pre- or postsynaptically for normal synaptic growth regulation, we expressed a *gef26* cDNA transgene (*UAS-gef26*) in *gef26* ^*6*^ */Df* mutants under the control of tissue-specific GAL4 drivers. Expression of *UAS-gef26* using a neuronal driver (*C155-GAL4*) fully rescued the NMJ growth defect of *gef26* mutants (Fig. 1b). In contrast, expression of *UAS-gef26* in all somatic muscles using the *BG57-GAL4* driver failed to rescue the NMJ growth defect (Fig. 1b), suggesting that Gef26 functions presynaptically to restrain synaptic growth at the NMJ.

Additional evidence for a presynaptic requirement for Gef26 was provided by assessment of the effect of RNA interference (RNAi)-mediated knockdown of Gef26 expression. Neuronal expression of a dsRNA-fragment of *gef26* (*UAS-gef26* ^*RNAi*^) using *C155-GAL4* increased both bouton number and satellite bouton number and mimicked the *gef26* loss-of-function mutation, whereas muscular expression of the same dsRNA using *BG57-GAL4* had no effect (Additional file 2: Figure S1a, b; Additional file 3: Table S2). This result supports the notion that Gef26 acts in presynaptic neurons to restrain synaptic growth at the NMJ.

We further characterized satellite boutons at *gef26* mutant NMJs using several synaptic markers. Satellite boutons contained the active zone antigen NC82 and the synaptic vesicle marker cysteine-string protein (CSP) (Additional file 2: Figure S1c, d). In addition, satellite boutons were found to recruit the subsynaptic reticulum (SSR) marker discs-large (Dlg). Finally, NC82 in satellite boutons was nicely juxtaposed to the essential glutamate receptor subunit GluRIIC (Additional file 2: Figure S1e, f). Thus, satellite boutons in *gef26* mutants display the anatomical hallmarks of functional synapses.

### Gef26 acts through Rap1 to regulate synaptic growth

**Fig. 2:**
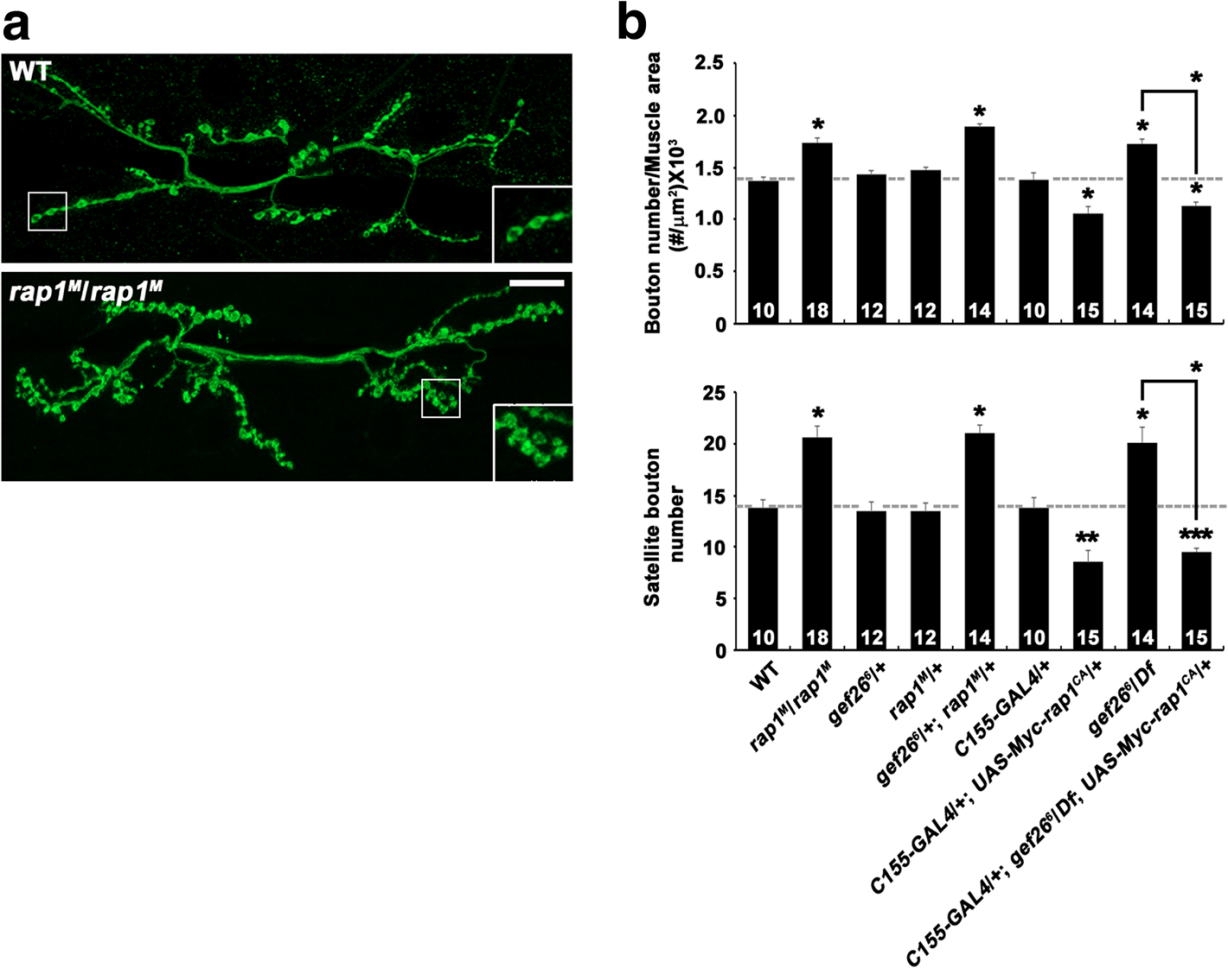
*gef26* interacts genetically with *rap1* at the NMJ. **a** Confocal images of anti-HRP-labeled NMJ 6/7 in wild-type and *rap1* ^*M*^/*rap1* ^*M*^ third-instar larvae. Scale bar, 20 μm. **b** Quantification of total bouton number normalized to muscle surface area and satellite bouton number at NMJ 6/7 in wild-type, *rap1* ^*M*^/*rap1* ^*M*^, *gef26* ^*6*^/+, *rap1* ^*M*^/+, *gef26* ^*6*^/+; *rap1* ^*M*^/+, *C155-GAL4*/+, *C155-GAL4*/+; *UAS-Myc-rap1* ^*CA*^/+, *gef26* ^*6*^/*Df*, and *C155-GAL4*/+; *gef26* ^*6*^/*Df*; *UAS-Myc-rap1* ^*CA*^/*+* backgrounds. The number of NMJs analyzed is indicated in each bar. Data are expressed as mean ± SEM. All comparisons are made with wild-type (*P < 0.001; **P < 0.01; ***P < 0.05)

Since Gef26 acts via Rap1 to mediate various developmental processes [15–17, 19, 22], we decided to investigate whether Rap1 is the major target for Gef26 in the regulation of synaptic growth. We began by investigating whether loss of *rap1* produces NMJ phenotypes similar to those caused by *gef26* loss-of-function mutations. For this purpose, we analyzed NMJ morphology in third instar larvae homozygous for the *rap1* ^*MI11950*^ allele (hereafter referred to as *rap1* ^*M*^) harboring a Minos element within the *rap1* gene. Compared with wild-type controls, both overall bouton number and satellite bouton number in *rap1* ^*M*^ mutants were significantly increased (Fig. 2a, b; Additional file 4: Table S3). To confirm the requirement for *rap1* in the proper regulation of synaptic growth, we also examined NMJ morphology in third instar larvae expressing *rap1* dsRNA (*UAS-rap1* ^*RNAi*^) under the control of *C155-GAL4*. This genetic manipulation significantly increased overall bouton number and satellite bouton number (Additional file 5: Figure S2a, b; Additional file 6: Table S4). In contrast, muscular expression of *UAS-rap1* ^*RNAi*^ did not noticeably alter NMJ morphology (Additional file 5: Figure S2a, b; Additional file 6: Table S4). Thus, loss of presynaptic *rap1* produces *gef26*-like phenotypes at the NMJ.

Next, we assayed the transheterozygous interaction between *gef26* and *rap1* during synaptic growth. Heterozygous *gef26* ^*6*^/+ or *rap1* ^*M*^/+ larvae displayed normal NMJ morphology. However, overall bouton number and satellite bouton number were both significantly increased in transheterozygous *gef26* ^*6*^/+; *rap1* ^*M*^/+ larvae compared with single *gef26* ^*6*^/+ or *rap1* ^*M*^/+ heterozygotes (Fig. 2b). This type of genetic interaction suggests that Gef26 and Rap1 function in the same pathway.

Finally, we explored the epistatic relationship between *gef26* and *rap1*. Neuronal overexpression of dominant-active Rap1-Q63E (*UAS-rap1* ^*CA*^) using *C155-GAL4* produced an NMJ undergrowth phenotype with fewer synaptic boutons (Fig. 2b). Importantly, neuronal overexpression of *UAS-rap1* ^*CA*^ was able to induce a similar phenotype even in the *gef26* ^*6*^ */Df* background (Fig. 2b), indicating that the overactivity of Rap1 completely suppresses the synaptic overgrowth in *gef26* mutants. These results suggest that Gef26 acts upstream of Rap1 to restrain synaptic growth at the NMJ.

### Gef26 and Rap1 regulate synaptic growth via inhibition of BMP signaling

**Fig. 3:**
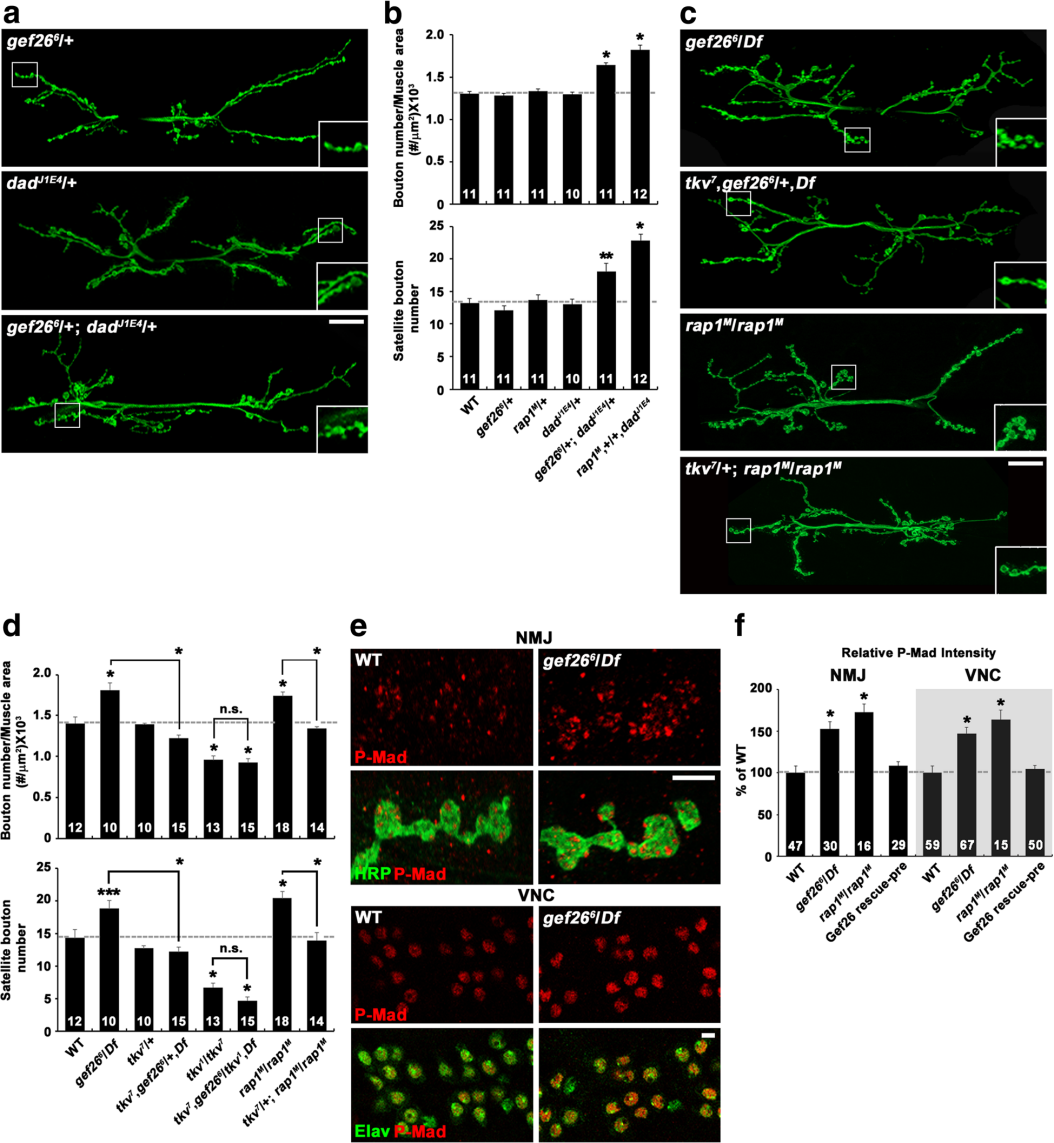
Gef26 and Rap1 inhibit BMP signaling to restrain synaptic growth. **a-d** *gef26*/*rap1* interacts with BMP signaling pathway components. **a** and **b** Transheterozygous interactions between *gef26* or *rap1* and *dad*. **a** Confocal images of anti-HRP-labeled NMJ 6/7 in *gef26* ^*6*^/+, *dad* ^*J1E4*^/+, and *gef26* ^*6*^/+; *dad* ^*J1E4*^/+ third-instar larvae. Scale bar, 20 μm. **b** Quantification of total bouton number and satellite bouton number at NMJ 6/7 in the following genotypes: wild-type, *gef26* ^*6*^/+, *rap1* ^*M*^/+, *dad* ^*J1E4*^/+, *gef26* ^*6*^/+; *dad* ^*J1E4*^/+, and *rap1* ^*M*^, +/+,dad^J1E4^. **c** and **d** Synaptic overgrowth in *gef26* and *rap1* depends on BMP signaling. **c** Confocal images of anti-HRP-labeled NMJ 6/7 in *gef26* ^*6*^/*Df*, *tkv* ^*7*^,*gef26* ^*6*^/+,*Df*, *rap1* ^*M*^/*rap1* ^*M*^, and *tkv* ^*7*^/+; *rap1* ^*M*^/*rap1* ^*M*^ third-instar larvae. Scale bar, 20 μm. **d** Quantification of total bouton number and satellite bouton number at NMJ 6/7 in the following genotypes: wild-type, *gef26* ^*6*^/*Df*, *tkv* ^*7*^/+, *tkv* ^*7*^,*gef26* ^*6*^/+,*Df*, *tkv* ^*1*^/*tkv* ^*7*^, *tkv* ^*7*^,*gef26* ^*6*^/*tkv* ^*1*^,*Df*, *rap1* ^*M*^/*rap1* ^*M*^, and *tkv* ^*7*^/+; *rap1* ^*M*^/*rap1* ^*M*^. Note that synaptic overgrowth in *gef26* and *rap1* mutants is significantly suppressed by loss of one copy of *tkv*. **e** and **f** Levels of pMad are increased in *gef26* and *rap1* mutants. **e** Confocal images of NMJ 6/7 and ventral nerve cord (VNC) labeled with anti-P-Mad and anti-HRP or anti-Elav in wild-type and *gef26* ^*6*^/*Df* third-instar larvae. Scale bars, 5 μm. **f** Quantification of the ratio of the average levels of P-Mad to HRP or Elav. Genotypes include wild-type, *gef26* ^*6*^/*Df*, *rap1* ^*M*^/*rap1* ^*M*^, and *C155-GAL4*/+; *gef26* ^*6*^/*Df*; *UAS-gef26*/+ (Gef26 rescue-pre). The number of NMJs or VNCs analyzed is indicated in each bar. Data are expressed as mean ± SEM. All comparisons are made with wild-type unless otherwise indicated (*P < 0.001; **P < 0.01; ***P < 0.05; n.s., not significant)

Previous studies have identified Gbb as a key retrograde signal that stimulates synaptic growth at the NMJ [4–7, 23]. Consistently, elevation of BMP signaling, which can be achieved by either presynaptic overexpression of a dominantly active Tkv receptor or loss of the inhibitory Smad Daughters against decapentaplegic (Dad), causes synaptic overgrowth with excessive satellite bouton formation [9, 10], recapitulating phenotypes exhibited by *gef26* or *rap1* mutants. Therefore, we wondered whether Gef26 and Rap1 might regulate synaptic growth by inhibiting BMP signaling. To test this possibility, we first examined the transheterozygous interaction between *gef26* or *rap1* and *dad* at the NMJ. Like *gef26* ^*6*^/+ and *rap1* ^*M*^/+ larvae, heterozygous *dad* ^*J1E4*^/+ larvae displayed normal NMJ morphology (Fig. 3a, b; Additional file 7: Table S5). In contrast, both overall bouton number and satellite bouton number were significantly increased in transheterozygous *gef26* ^*6*^/+; *dad* ^*J1E4*^/+ and *rap1* ^*M*^,+/+,*dad* ^*J1E4*^ larvae compared with wild-type controls (Fig. 3a, b), suggesting a functional link between Gef26/Rap1 and the BMP signaling pathway during synaptic growth.

We next examined whether synaptic overgrowth in *gef26* or *rap1* mutants depends on BMP signaling. Heterozygosity for the BMP receptor gene *tkv* (*tkv* ^*7*^/+), which had no effect on NMJ morphology in a wild-type background, suppressed synaptic overgrowth in *gef26* ^*6*^ */Df* or *rap1* ^*M*^/*rap1* ^*M*^ mutants (Fig. 3c, d; Additional file 7: Table S5). Moreover, removal of both copies of *tkv* (*tkv* ^*1*^ */tkv* ^*7*^) in the *gef26* ^*6*^ */Df* background caused a synaptic undergrowth phenotype, which was similar to that of *tkv* ^*1*^ */tkv* ^*7*^ mutants (Fig. 3c, d). Thus, BMP signaling is necessary for synaptic overgrowth in *gef26* or *rap1* mutants.

Finally, we directly tested the role of Gef26/Rap1 in inhibiting BMP signaling by assaying P-Mad levels in *gef26* and *rap1* mutants. P-Mad accumulation at NMJ synapses and in the nuclei of ventral nerve cord (VNC) motoneurons was significantly increased in *gef26* ^*6*^ */Df* or *rap1* ^*M*^/*rap1* ^*M*^ larvae compared with wild-type controls (Fig. 3e, f). Neuronal expression of *UAS-gef26* in *gef26* ^*6*^ */Df* mutants was capable of reversing the increase of P-Mad in motoneurons (Fig. 3f), establishing the roles of Gef26 and Rap1 as negative regulators of BMP signaling. These results support a model in which Gef26 and Rap1 restrain synaptic growth by inhibiting BMP signaling.

### Gef26 and Rap1 control BMP-dependent synaptic growth by regulating *Drosophila fragile X mental retardation 1* (*dfmr1*) expression and microtubule stability

**Fig. 4:**
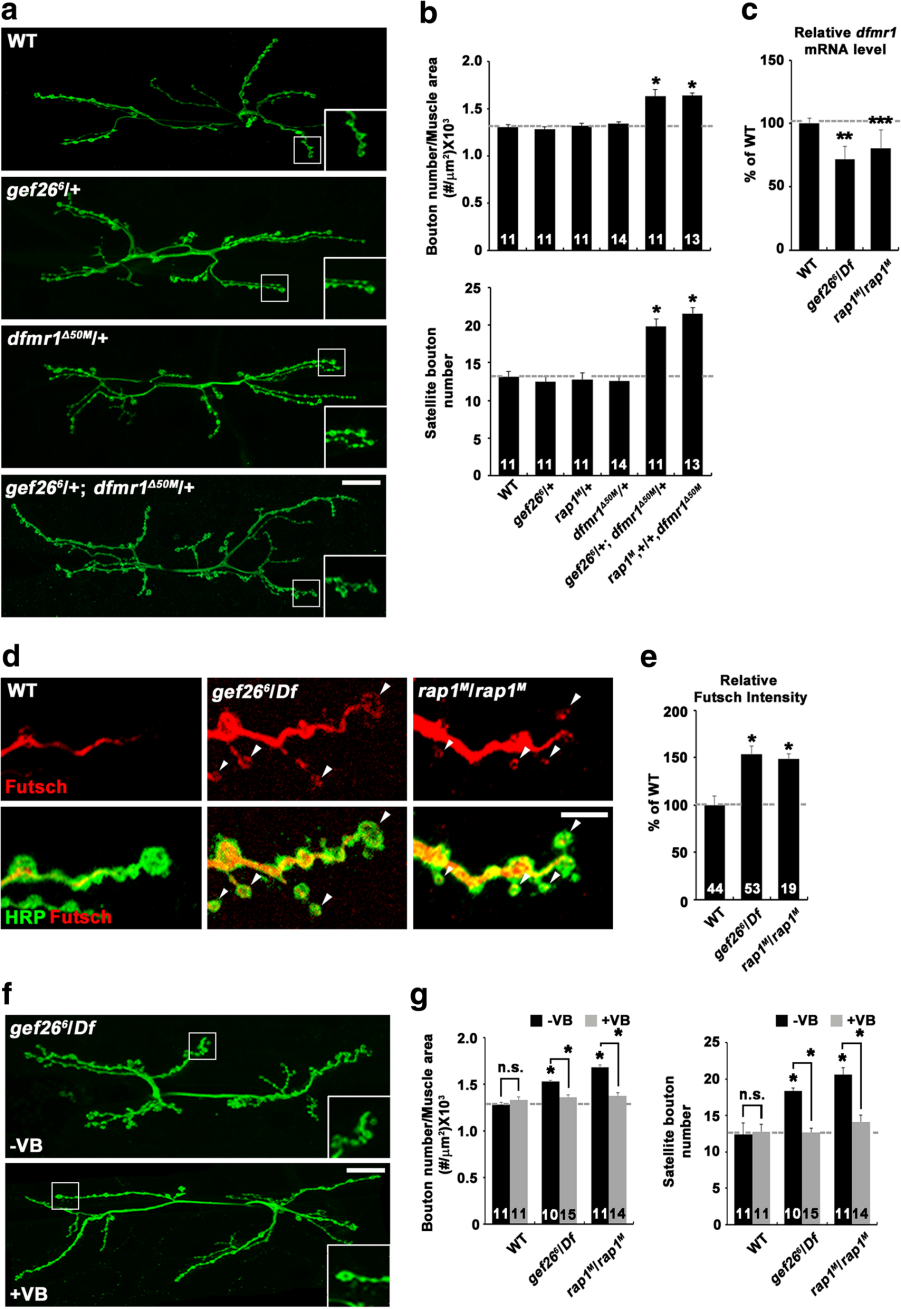
Altered *dfmr1* expression and microtubule stability cause synaptic overgrowth in *gef26* and *rap1* mutants. **a** and **b** Transheterozygous interactions between *gef26* or *rap1* and *dfmr1*. **a** Confocal images of anti-HRP-labeled NMJ 6/7 in wild-type, *gef26* ^*6*^/+, *dfmr1* ^*Δ50M*^/+, and *gef26* ^*6*^/+; *dfmr1* ^*Δ50M*^ */+* third-instar larvae. Scale bar, 20 μm. **b** Quantification of total bouton number and satellite bouton number at NMJ 6/7 in the following genotypes: wild-type, *gef26* ^*6*^/+, *rap1* ^*M*^/+, *dfmr1* ^*Δ50M*^/+, *gef26* ^*6*^/+; *dfmr1* ^*Δ50M*^ */+*, and *rap1* ^*M*^,+/+,*dfmr1* ^*Δ50M*^. **c** Quantification of *dfmr1* RNA levels using quantitative real-time PCR in the CNS of wild-type, *gef26* ^*6*^/*Df*, and *rap1* ^*M*^/*rap1* ^*M*^ third-instar larvae. *rp49* was used as an internal control. **d** and **e** Levels of synaptic Futsch are increased in *gef26* and *rap1* mutants. **d** Confocal images of anti-Futsch and anti-HRP staining from NMJ 6/7 of wild-type, *gef26* ^*6*^/*Df*, and *rap1* ^*M*^/*rap1* ^*M*^ third-instar larvae. Arrowheads indicate Futsch-positive terminal loops. Scale bar, 5 μm. **e** Quantification of the ratio of the average anti-Futsch to anti-HRP staining intensities. **f** and **g** Synaptic overgrowth in *gef26* and *rap1* mutants is suppressed by vinblastine administration. **f** Confocal images of NMJ 6/7 immunostained with anti-HRP are shown for *gef26* ^*6*^/*Df* mutants raised in the absence (-VB) or presence (+VB) of 1 μM vinblastine. Scale bar, 20 μm. **g** Quantification of total bouton number and satellite bouton number at NMJ 6/7 in the indicated genotypes. The number of NMJs analyzed is indicated in each bar. Data are expressed as mean ± SEM. All comparisons are made with wild-type unless otherwise indicated (*P < 0.001; **P < 0.01; ***P < 0.05; n.s., not significant)

At the *Drosophila* NMJ, BMP signaling has been shown to repress the expression of the *dfmr1* gene [9]. The *dfmr1* product (dFMRP) in turn negatively regulates the expression of the microtubule-associated protein 1B (MAP1B) Futsch [24], which promotes synaptic growth by stabilizing synaptic microtubules [25]. Therefore, we hypothesized that Gef26/Rap1 might control synaptic growth by regulating microtubule stability via the dFMRP-Futsch pathway. To test the involvement of dFMRP in Gef26/Rap1-dependent regulation of synaptic growth, we first examined the transheterozygous interaction between *gef26* or *rap1* and *dfmr1* at the NMJ. Total bouton number and satellite bouton number were significantly higher in transheterozygous *gef26* ^*6*^/+; *dfmr1* ^*Δ50M*^/+ and *rap1* ^*M*^, +/+,*dfmr1* ^Δ50M^ larvae than in wild-type controls, although the single heterozygotes displayed normal synaptic growth (Fig. 4a, b; Additional file 8: Table S6). In a subsequent experiment, we directly tested whether loss of Gef26 or Rap1 alters *dfmr1* expression. Levels of *dfmr1* mRNA were significantly lower in *gef26* and *rap1* mutants than in wild-type controls, as demonstrated by quantitative real-time PCR (Fig. 4c). Given the roles of Gef26 and Rap1 in inhibiting BMP signaling, these results imply that Gef26/Rap1 restrains synaptic growth by relieving BMP-dependent repression of *dfmr1* transcription.

Next, we investigated whether *gef26* and *rap1* mutants affect synaptic Futsch levels. In wild-type NMJs, Futsch was detected as a filamentous bundle occupying the center of the presynaptic terminals. However, Futsch staining was fainter or not detectable in newly formed or terminal boutons. Futsch immunoreactivity was significantly increased in *gef26* and *rap1* mutant axons compared with wild-type controls (Fig. 4d, e). In addition, the number of terminal boutons with Futsch immunoreactivity (visualized as a looped or punctate structure) was significantly higher in *gef26* ^*6*^ */Df* or *rap1* ^*M*^/*rap1* ^*M*^ mutants (Fig. 4d, arrowheads). These results indicate that Gef26 and Rap1 function to limit presynaptic Futsch level.

Futsch reliably labels microtubules in presynaptic motor terminals [25]. Therefore, the above results suggest the involvement of microtubule stability in Gef26/Rap1-mediated regulation of synaptic growth. To directly test this possibility, we assayed the extent of synaptic growth in *gef26* and *rap1* mutants fed vinblastine, a microtubule-severing drug [26]. When vinblastine was fed at a low concentration (1 μM) that did not affect synaptic growth, it completely suppressed the synaptic overgrowth phenotype of *gef26* ^*6*^ */Df* or *rap1* ^*M*^/*rap1* ^*M*^ larvae (Fig. 4f, g; Additional file 8: Table S6). These results support the idea that Gef26/Rap1 controls synaptic growth by regulating microtubule stability via the Futsch pathway.

### Gef26 regulates the endocytic internalization of the BMP receptors Tkv and Wit

**Fig. 5:**
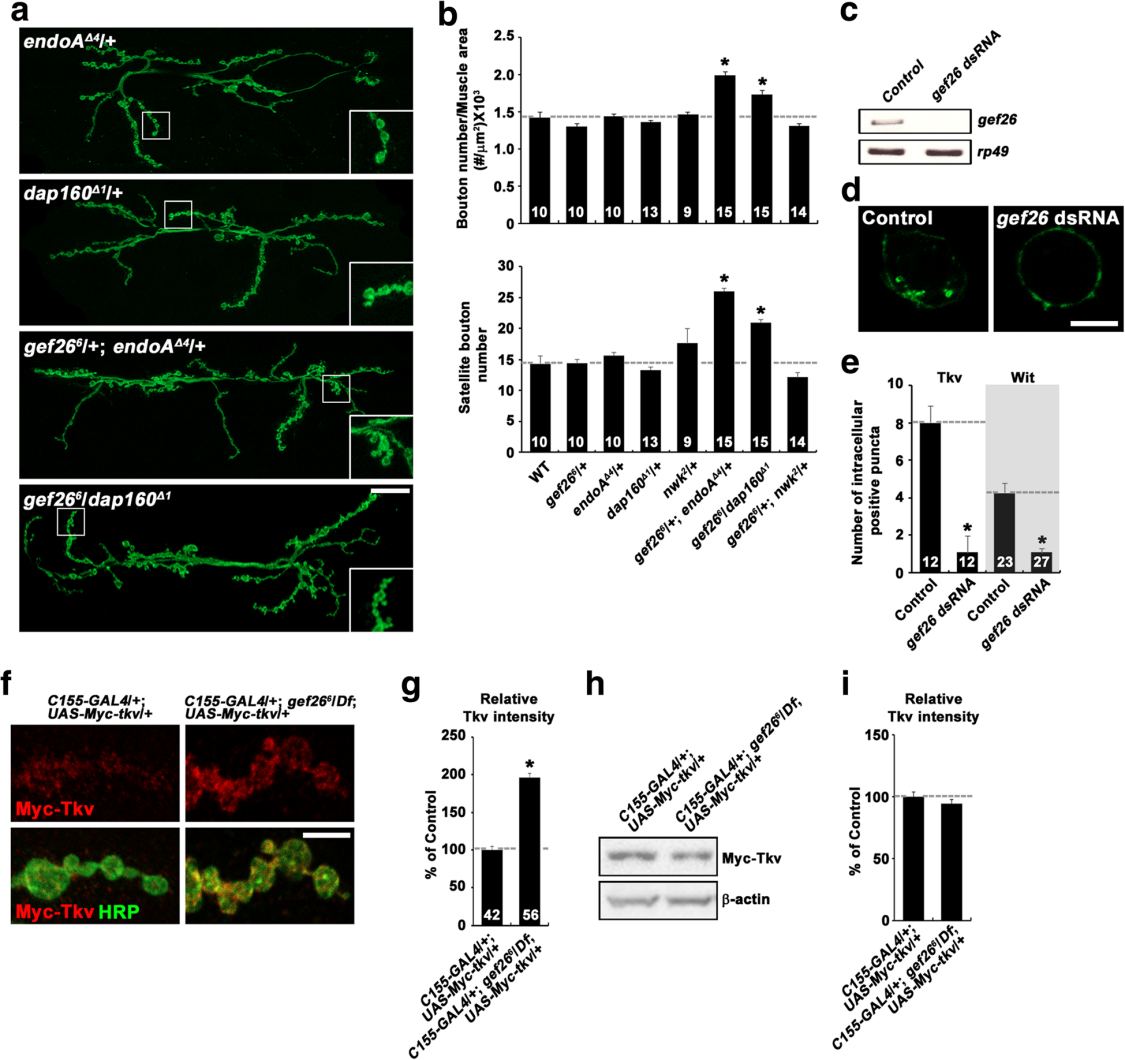
Gef26 regulates endocytic internalization and surface expression of BMP receptors. **a** and **b** *gef26* interacts with endocytic mutations during synaptic growth. **a** Confocal images of anti-HRP-labeled NMJ 6/7 in *endoA* ^*Δ4*^/+, *dap160* ^*Δ1*^/+, *gef26* ^*6*^/+; *endoA* ^*Δ4*^/+, and *gef26* ^*6*^/*dap160* ^*Δ1*^ third-instar larvae. Scale bar, 20 μm. **b** Quantification of total bouton number and satellite bouton number at NMJ 6/7 in the following genotypes: wild-type, *gef26* ^*6*^/+, *endoA* ^*Δ4*^/+, *dap160* ^*Δ1*^/+, *nwk* ^*2*^/+, *gef26* ^*6*^/+; *endoA* ^*Δ4*^/+, *gef26* ^*6*^/*dap160* ^*Δ1*^, and *gef26* ^*6*^/+; *nwk* ^*2*^/+. **c-e** Gef26 is required for endocytic internalization of surface BMP receptors. BG2-c2 cells were transfected with *pAc-Myc-tkv-Flag* or *pAc-Myc-wit-Flag* in the absence (control) and presence of *gef26* dsRNA. Live control and Gef26-depleted cells were prelabeled with anti-Myc (green) at 4 °C, followed by incubation at 25 °C for 10 min to allow internalization of the labeled surface receptors. After fixation and permeabilization, cells were sequentially stained with anti-Flag (red) and fluorescently-labeled secondary antibodies. **c** Reverse transcription (RT)-PCR analysis to confirm knockdown efficiency of Gef26. **d** Single confocal sections through the middle of control and *gef26*-knockdown cells are shown for the green channel only. Scale bar, 5 μm. **e** Quantification of the number of intracellular Myc-positive puncta per cell. Only cells with similar Flag signal (red) intensities were analyzed. **f** and **g** Steady-state levels of surface Tkv are increased at the NMJ of *gef26* mutants. **f** Representative confocal images of NMJ 6/7 in *C155-GAL4*/+; *UAS-Myc-tkv*/+ and *C155-GAL4*/+; *gef26* ^*6*^ */Df*; *UAS-Myc-tkv*/+ larvae. NMJ preparations were sequentially stained with anti-Myc (red) and anti-HRP (green) under nonpermeant and permeant conditions. Scale bar, 5 μm. **g** Quantification of the ratio of surface Myc-Tkv to HRP fluorescence intensities. **h** and **i** Transgenic expression of Myc-Tkv in neurons is not altered by loss of Gef26. **h** Western blot of central nervous system (CNS) extracts from *C155-GAL4*/+; *UAS-Myc-tkv*/+ and *C155-GAL4*/+; *gef26* ^*6*^ */Df*; *UAS-Myc-tkv*/+ larvae. The blot was probed with anti-Myc and anti-β-actin. **i** Quantitative analysis of three independent blots by densitometric measurements. For each sample, the band intensity of Myc-Tkv was normalized to that of β-actin. The number of NMJs (**b**), cells (**e**), or synaptic boutons (**g**) analyzed is indicated in each bar. Data are expressed as mean ± SEM. *P < 0.001

We next attempted to determine how Gef26 attenuates BMP signaling. Mutations disrupting endocytosis, including *endophilin* (*endo*) and *dap160*, increase presynaptic P-Mad levels at the NMJ along with simultaneous synaptic overgrowth and the formation of excessive satellite boutons [10, 13, 27], suggesting that endocytosis of surface BMP receptors is an important mechanism to inhibit BMP-dependent synaptic growth. Since a similar phenotype was observed in *gef26* mutants, we wondered if Gef26 regulates BMP signaling through endocytosis. To test this possibility, we first investigated genetic interactions between *gef26* and mutations in endocytic genes. In heterozygous *gef26* ^*6*^/+, *endoA* ^*Δ4*^/+, and *dap160* ^*Δ1*^/+ larvae, total bouton number and satellite bouton number were at wild-type levels (Fig. 5a, b; Additional file 9: Table S7). In sharp contrast, both parameters were significantly increased in transheterozygous *gef26* ^*6*^/+; *endoA* ^*Δ4*^/+, or *gef26* ^*6*^/*dap160* ^*Δ1*^ larvae (Fig. 5a, b), raising the possibility that Gef26 regulates BMP-dependent synaptic growth through an endocytic mechanism. It has been proposed that Dap160 interacts with the endosomal protein Nervous wreck (Nwk) to negatively regulate synaptic growth [10, 28]. However, total bouton number and satellite bouton number were normal in transheterozygous *gef26* ^*6*^/+; *nwk* ^*2*^/+ larvae (Fig. 5b), suggesting that Gef26 and Nwk regulate BMP signaling through distinct pathways.

We then examined the impact of *gef26* knockdown on the endocytic internalization of BMP receptors in neuronal BG2-c2 cells. We transiently transfected a Myc-Tkv-Flag or Myc-Wit-Flag construct into control or *gef26*-knockdown cells (Fig. 5c) and prelabeled the cells with an anti-Myc antibody at 4 °C. We then initiated endocytosis by incubating the cells at 25 °C for 10 min and visualized the internalization of the labeled surface receptors by Myc staining. Total Myc-Tkv-Flag or Myc-Wit-Flag was also monitored by staining for the intracellular Flag-tag after cellular permeabilization. In controls cells, we observed several Myc-Tkv-Flag- or Myc-Wit-Flag-positive intracellular puncta (Fig. 5d; data not shown). Importantly, when examined in only cells with similar fluorescence intensities of Flag staining, the number of intracellular Myc-Tkv-Flag- or Myc-Wit-Flag-positive puncta per cell was dramatically reduced in *gef26*-knockdown cells (Fig. 5d, e), suggesting that Gef26 is required for the endocytic internalization of BMP receptors.

Next, we determined the impact of *gef26* loss-of-function on the levels of surface Tkv at the NMJ. To do this, we expressed *UAS-Myc-tkv* in wild-type and *gef26* mutants using *C155-GAL4*. Surface Myc-Tkv and total HRP were measured by sequential staining with anti-Myc and anti-HRP antibodies under nonpermeant and permeant conditions, respectively. The ratio of Myc-Tkv signal to HRP signal intensity was significantly increased in *gef26* compared with wild-type NMJs (Fig. 5f, g). Levels of Myc-Tkv expression were not significantly different between wild-type and *gef26* ^*6*^/*Df* animals (Fig. 5h, i). These data support a role for Gef26 in endocytic internalization of the Tkv receptor.

Given the role of Gef26 in BMP receptor internalization, we examined whether synaptic vesicle endocytosis is affected in *gef26* mutant NMJs. We stimulated third instar fillets with 90 mM K^+^ in the presence of the styryl dye FM1–43FX. During a 1-min labeling period, dye uptake into synaptic boutons was not significantly different between wild-type and *gef26* ^*6*^ */Df* mutant animals (Additional file 10: Figure S3a, b). This result indicates that loss of Gef26 does not grossly affect endocytosis at the presynaptic terminal of the NMJ.

### Gef26/Rap1 regulation of BMP signaling is essential for neuronal survival in the adult brain

**Fig. 6:**
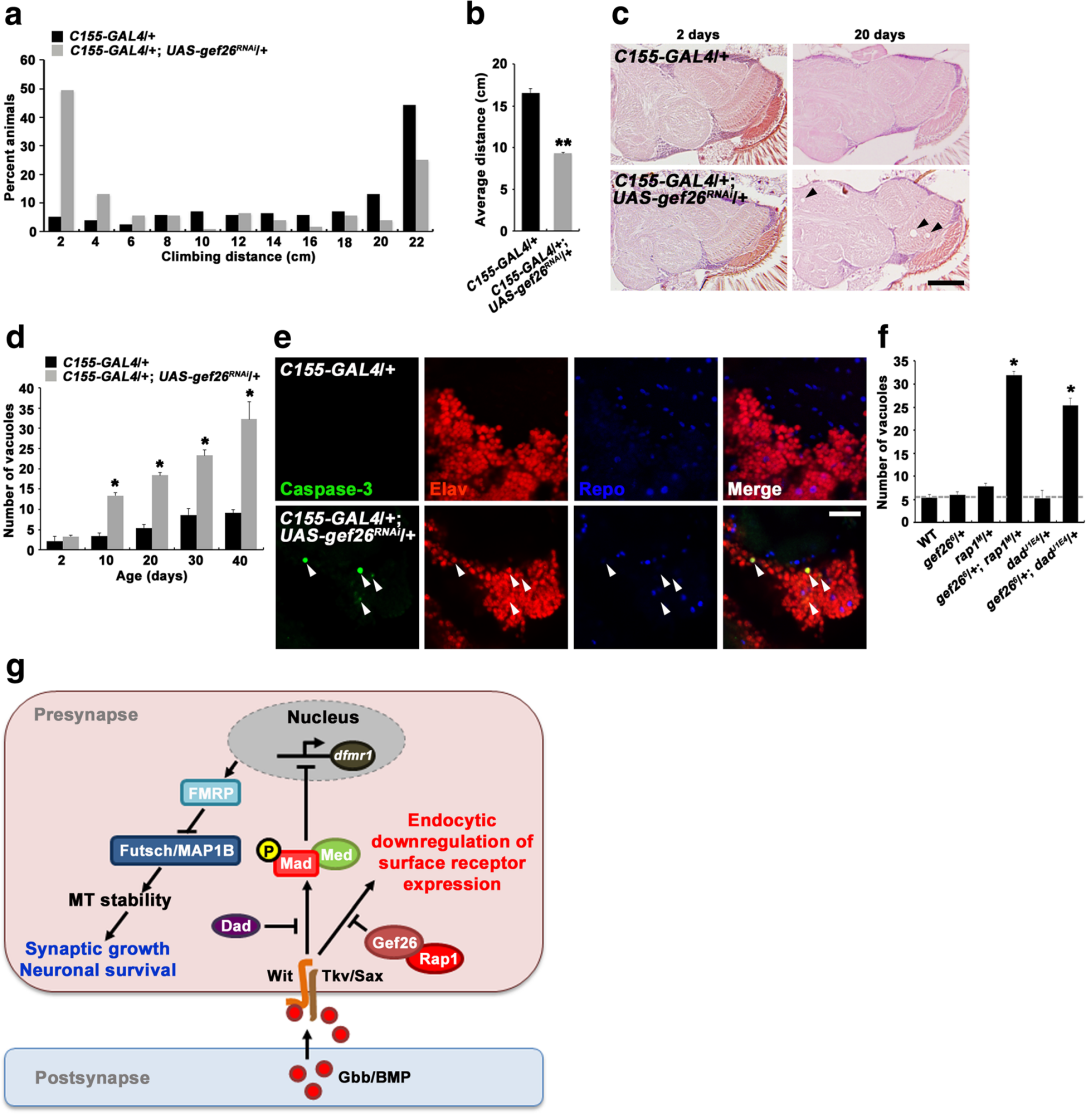
Loss of Gef26 induces motor dysfunction and progressive brain neurodegeneration in the adult fly. **a** and **b** Adult locomotor activity is impaired by neuron-specific Gef26 knockdown. **a** Distribution of the distance climbed by 20-day-old *C155-GAL4*/+ and *C155-GAL4*/+; *UAS-gef26* ^*RNAi*^/+ flies over a 30 s period. **b** Quantification of average climbing distance. **c** and **d** Neuron-specific Gef26 knockdown causes brain neurodegeneration in an age-dependent manner. **c** Frontal brain sections (5 μm) stained with H&E are shown for *C155-GAL4*/+ and *C155-GAL4*/+; *UAS-gef26* ^*RNAi*^/+ flies at 2 or 20 days of age. Note the presence of numerous vacuoles (arrowheads) in the brain of 20-day-old *C155-GAL4*/+; *UAS-gef26* ^*RNAi*^/+ flies. Scale bar, 20 μm. **d** Quantification of vacuoles with a diameter greater than 5 μm in *C155-GAL4*/+ and *C155-GAL4*/+; *UAS-gef26* ^*RNAi*^/+ brains at different ages. *n* > 10. **e** Confocal sections of 20-day-old *C155-GAL4*/+ and *C155-GAL4*/+; *UAS-gef26* ^*RNAi*^/+ brains stained with anti-caspase-3 (green), anti-Elav (red), and anti-Repo (blue). Anti-caspase-3 signals overlaps with the neuronal cell marker anti-Elav (arrowheads) but not with the glial cell marker anti-Repo. Scale bar, 20 μm. **f** Quantification of brain vacuolization in 20-day-old wild-type (*n* = 5), *gef26* ^*6*^/+ (*n* = 5), *rap1* ^*M*^/+ (*n* = 6), *gef26* ^*6*^/+; *rap1* ^*M*^/+ (*n* = 5), *dad* ^*J1E4*^/+ (*n* = 5), and *gef26* ^*6*^/+; *dad* ^*J1E4*^/+ (*n* = 6) flies. **g** Model for Gef26/Rap1 regulation of BMP-dependent synaptic growth and neuronal survival

Overactivation of BMP signaling in the adult *Drosophila* brain induces age-dependent progressive motor dysfunction and neurodegeneration [9]. Since we established the role of Gef26 in downregulating BMP signaling at the NMJ, we investigated whether *gef26* knockdown induces adult phenotypes similar to elevated BMP signaling. We first assayed the locomotor performance of *C155-GAL4*/+; *UAS-gef26* ^*RNAi*^/+ flies in a geotactic climbing assay. Compared with age-matched *C155-GAL4*/+ controls, 20-day-old *C155-GAL4*/+; *UAS-gef26* ^*RNAi*^/+ flies displayed a significantly reduced climbing response within 30 s (*C155-GAL4*/+: 16.56 ± 0.57 cm, *C155-GAL4*/+; *UAS-gef26* ^*RNAi*^/+: 9.33 ± 0.11 cm, *P* < 0.001; Fig. 6a, b).

We then investigated whether knockdown of *gef26* expression is associated with neurodegeneration by examining histological sections of adult brains. At 2 days after eclosion, *C155-GAL4*/+; *UAS-gef26* ^*RNAi*^/+ brains had normal anatomical and histological organization (Fig. 6c). However, aged *C155-GAL4*/+; *UAS-gef26* ^*RNAi*^/+ brains exhibited progressive vacuolization (Fig. 6c, arrowheads), which is a hallmark of neurodegeneration in the *Drosophila* brain [29]. Vacuolization progressed at a much slower rate in *C155-GAL4*/+ control brains (Fig. 6d). To further characterize neurodegeneration, we performed caspase-3 and TUNEL staining on 20-day-old brains. Caspase-3- or TUNEL-positive cells were detected in *C155-GAL4*/+; *UAS-gef26* ^*RNAi*^/+ brains, but not in *C155-GAL4*/+ control brains (Fig. 6e; Additional file 11: Figure S4a, b). In addition, TUNEL staining revealed that neuronal knockdown of *gef26* induces cell death in an age-dependent, progressive manner (Additional file 11: Figure S4b). Importantly, anti-caspase-3 signals overlapped with the neuronal marker anti-Elav, but not with the glial marker anti-Repo (Fig. 6e, arrowheads). Together, these results indicate that Gef26 activity is essential for neuronal survival in the adult brain.

Finally, we investigated whether Gef26 collaborates with Rap1 and the BMP pathway to maintain normal locomotor ability and neuronal survival. To this end, we first examined transheterozygous combinations of *gef26* and *rap1* or *dad* with respect to locomotor dysfunction. At 20 days of age, transheterozygous *gef26* ^*6*^/+; *rap1* ^*M*^/+ and *gef26* ^*6*^/+; *dad* ^*J1E4*^/+ flies displayed mildly reduced climbing response compared with age-matched *gef26* ^*6*^/+, *rap1* ^*M*^/+, or *dad* ^*J1E4*^/+ flies (data not shown). However, these transheterozygous flies at 30 days of age exhibited severely reduced climbing ability (Additional file 11: Figure S4c, d). We also examined transheterozygous interactions between *gef26* and *rap1* or *dad* with respect to brain neurodegeneration. At 20 days of age, heterozygous *gef26* ^*6*^/+, *rap1* ^*M*^/+, or *dad* ^*J1E4*^/+ flies were not distinguishable from wild-type controls with respect to the total number of vacuoles (Fig. 6f). In sharp contrast, there was a significant vacuolization in the brains of transheterozygous *gef26* ^*6*^/+; *rap1* ^*M*^/+ or *gef26* ^*6*^/+; *dad* ^*J1E4*^/+ flies (Fig. 6f), supporting a functional link between Gef26, Rap1, and the BMP signaling pathway in the regulation of neuronal survival in the adult brain.

## Discussion

In the mammalian nervous system, PDZ-GEF1 (also called RAPGEF2) and its downstream GTPase Rap1 play an important role in homeostatic synaptic plasticity by decreasing the density of dendritic spines [30], the primary postsynaptic compartment for excitatory synapses. However, the presynaptic function of these molecules has not been addressed. In this study, we identified the *Drosophila* homologs (Gef26 and Rap1, respectively) as new presynaptic regulators of BMP-dependent synaptic growth. First, *gef26* and *rap1* mutant NMJs display a distinctive phenotype of excessive satellite bouton formation, recapitulating synaptic defects induced by elevated BMP signaling [9, 10, 12]. Second, genetic epistasis analysis and several independent genetic experiments demonstrate that Gef26 acts upstream of Rap1 at presynaptic NMJ terminals. Third, synaptic overgrowth in *gef26* and *rap1* mutants depends on the level of BMP signaling. Fourth, *gef26* and *rap1* mutants show a significant increase in the level of P-Mad, the readout of BMP signaling. Fifth, Gef26 and Rap1 negatively regulate the stability of presynaptic microtubules via dFMRP, which is known to be a major target for BMP signaling in the regulation of synaptic growth [9]. Based on these data, we propose that the presynaptic GEF26-Rap1 pathway regulates synaptic growth by modulating microtubule stability via the BMP-dFMRP pathway.

How might Gef26 regulate BMP signaling? Increasing evidence suggests that endocytosis of surface BMP receptors is a key mechanism of signal attenuation at presynaptic NMJ terminals. In support of this notion, our data imply that Gef26 inhibits BMP signaling by regulating the endocytic internalization of its receptor(s). *gef26* displays transheterozygous interactions with mutations disrupting endocytosis (i.e., *dap160* and *endoA*) during synaptic growth. In addition, *gef26* mutant NMJs show an increase in the level of surface Tkv, supporting the role of Gef26 in receptor endocytosis. Most directly, we show that Gef26 facilitates the endocytic internalization of the BMP receptors Tkv and Wit in cultured cells. These findings imply a model in which Gef26 attenuate BMP signaling through facilitating endocytosis of BMP receptors (Fig. 6g).

Elevated BMP signaling has been implicated in the pathogenesis of hereditary spastic paraplegia (HSP), a group of neurodegenerative motor disorders. In mammalian cells, several HSP proteins, including NIPA1, Spastin, and Spartin, have been shown to inhibit BMP signaling [31]. At the *Drosophila* NMJ, the NIPA1 homologue Spichthyin (Spict) and Spartin also inhibit BMP signaling to restrain synaptic growth [9, 11]. Importantly, it has now been demonstrated that elevation of BMP signaling in adult *spartin* flies causes progressive neurodegeneration and locomotor dysfunction [9]. Consistent with these studies and the proposed role of Gef26 as an inhibitor of BMP signaling, depletion of *gef26* in the adult fly induces neurodegeneration and locomotor function. Thus, the current study solidifies the notion that precise regulation of BMP signaling is critical for the maintenance of adult neurons. A future challenge will be to investigate whether PDZ-GEF1 and other human Gef26 homologues contribute to the maintenance of the human motor system and, if so, whether this neuroprotective role involves the regulation of retrograde BMP transsynaptic signaling.

A final point of interest is the mechanism of how the Gef26-Rap1 pathway facilitates BMP receptor endocytosis. In various experimental systems, Rap1 has been identified to regulate actin-driven cellular processes. For example, mammalian Rap1 promotes cell spreading by localizing the RacGEFs Vav2 and Tiam1 to sites of lamellipodia extension [32], which is driven by Rac-dependent actin polymerization. In addition, *Dictyostelium* Rap1 is also involved in chemotaxis by activating the Rac signaling pathway through RacGEF1 [33]. Since actin polymerization is known to provide mechanical forces required for multiple stages of endocytosis [34], it is tempting to speculate that Rap1 facilitates endocytosis by regulating actin polymerization through the RacGEF-Rac signaling pathway. Interestingly, the Rac signaling pathway has been implicated in the regulation of BMP-dependent synaptic growth at the *Drosophila* NMJ [35]. In future studies, it will be interesting to investigate the role of the Rac signaling pathway in Rap1-dependent endocytosis.

## Methods

### *Drosophila* stocks

Flies were maintained on standard medium at 25 °C. *w* ^*1118*^ was used as the wild-type control. *gef26* ^*6*^ was generously provided by S. Hou (National Institutes of Health National Cancer Institute, Frederick, MD, USA) [19], and *nwk* ^*2*^ was obtained from K. O’Connor-Giles (University of Wisconsin, Madison, WI, USA) [10]. *Df(2 L)BSC5* (a deficiency of the *gef26* locus), *rap1* ^*MI11950*^, *tkv* ^*1*^, *tkv* ^*7*^, *dad* ^*J1E4*^, *endoA* ^*Δ4*^, and *dap160* ^*Δ1*^ were obtained from the Bloomington Stock Center (Bloomington, IN, USA). Transgenic lines carrying *UAS-gef26*, *UAS-Myc-rap1* ^*CA*^, and *UAS-Myc-tkv* were generated in the *w* ^*1118*^ background using standard protocols. RNA interference (RNAi) lines PDZ-GEF^KK102612^ (referred to here as *UAS-gef26* ^*RNAi*^) and Rap1^KK107785^ (*UAS-rap1* ^*RNAi1*^) were obtained from the Vienna Drosophila Resources Center (Vienna, Austria). Another RNAi line Rap1^HMJ21898^ (*UAS-rap1* ^*RNAi2*^) was obtained from the Bloomington Stock Center. Neural and muscle-specific expression of UAS transgenes was achieved using *C155-GAL4* [36] and *BG57-GAL4* [37], respectively.

### Cell culture and transient transfection

*Drosophila* neuronal BG2-c2 cells were maintained at 25 °C in M3 medium (Sigma-Aldrich, St. Louis, MO, USA) supplemented with 10% heat-inactivated fetal bovine serum (Gibco, Carlsbad, CA, USA), 10 μg/ml insulin (Sigma-Aldrich), and penicillin/streptomycin. Cells were transfected in 12-well plates with 1 μg plasmid DNA in the presence or absence of 5 μg double-stranded RNA (dsRNA) using Cellfectin II (Invitrogen, Carlsbad, CA, USA).

### Molecular biology

Full-length cDNAs for *gef26* and *rap1* were obtained by reverse transcription PCR of total RNA extracted from *Drosophila* S2R+ cells and introduced into the pUAST or pUAST-Myc vector to generate *UAS-gef26* and *UAS-Myc-rap1*. For *UAS-Myc-rap1* ^*CA*^, glutamine 63 was mutated to glutamate by overlapping PCR using *UAS-Myc-rap1* (the template DNA) and the primers 5’-ATGGCCGTGAACTCCTCCGTACCC-3′ and 5’-TACGGAGGAGTTCACGGCCATGCG-3′ in combination with the BglII-Myc-linked primer 5’-GGGAGATCTGCCACCATGGAACAAAAACTCATCTCAGAAGAG-GATCTGATGCGTGAGTACAAAATC-3′ and the XbaI-linked primer 5’-GGGTCTAGATAGCAGAACACATAGGGAC-3′, respectively, and the assembled product was introduced into pUAST. For *pAc-Myc-tkv-Flag*, a full-length cDNA (clone ID: LD45557) for *tkv* (*CG1402*6) was obtained from the Drosophila Genomics Resource Center (Bloomington, IN, USA). The cDNA insert was PCR-amplified and then introduced into the pTOP Blunt V2 vector (Enzynomics, Daejeon, Republic of Korea). Myc and Flag epitope-tag sequences were introduced immediately downstream of the signal sequence and at the C-terminus of Tkv, respectively, by PCR-based mutagenesis. The resulting *Myc-tkv-Flag* insert was subcloned into the pAc5.1 vector. For *pAc-Myc-wit-Flag*, Flag epitope-tag sequence was introduced downstream of the *wit* sequence of *pAc-Myc-wit* [9] by PCR-based mutagenesis. The resulting *Myc-wit-Flag* fragment was re-introduced into the pAc5.1 vector.

To measure levels of *dfmr1* expression, total RNA was extracted from the third instar brain and ventral ganglion using the TRIsure kit (Bioline, Taunton, MA, USA) and reverse transcribed using the SuperScript III cDNA synthesis kit (Invitrogen). Quantitative real-time PCR reactions were performed using SYBR Select Master Mix (Applied Biosystems, Foster City, CA, USA) on an Applied Biosystems 7500 Real-Time PCR System. The mean Ct of triplicate reactions was used to determine relative expression of *dfmr1* using the 2^-*ΔΔ*CT^ method. Expression of *rp49* was used as the internal control. The primers used were: *dfmr1*, 5’-GGATCAGAACATACCACGTG-3′ and 5’-CTGGCAGCTATCGTGGAGGCG-3′; and *rp49*, 5’-CACCAGTCGGATCGATATGC-3′ and 5’-CACGTTGTGCACCAGGAACT-3′.

For RNA interference (RNAi) experiments in BG2-c2 cells, *gef26* dsRNA was produced by in vitro transcription of a DNA template containing T7 promoter sequences at both ends, as described previously [38]. The DNA template was produced by PCR from the *UAS-gef26* vector using primers containing a T7 promotor sequence followed by *gef26*-specific sequences: 5’-GTGGCCGGCTCTACCAGT-3′ and 5’-TGGTACGCGAGTCGAACG-3′.

### Western blot analysis

Larval CNS (the brain lobes and the ventral nerve cord) preparations were homogenized in ice-cold lysis buffer (25 mM Tris-HCl, pH 7.5, 150 mM NaCl, 0.5% Triton X-100, and protease inhibitors) and subjected to western blotting as described previously [20]. The following primary antibodies were used: anti-Myc (1:1000, Cell Signaling, Danvers, MA, USA) and anti-β-actin (1:1000, Sigma-Aldrich).

### BMPR internalization assay

BG2-c2 cells were transfected with *pAc-Myc-tkv-Flag* or *pAc-Myc-wit-Flag* in the presence or absence of *gef26* dsRNA. At 72 h post-transfection, live cells were incubated with an anti-Myc antibody (1:200, Cell Signaling) at 4 °C for 1 h to label Myc-Tkv-Flag or Myc-Wit-Flag proteins expressed on the cell surface, followed by incubation at 25 °C for 10 min to allow internalization of the labeled receptors. Cells were subsequently washed in an ice-cold acidic buffer (0.5 M NaCl, 0.2 M acetic acid, pH 4.0) for 15 min to remove any remaining bound anti-Myc antibody and fixed in PBS containing 4% formaldehyde for 10 min. Fixed cells were permeabilized in PBT-0.2 (PBS, 0.2% Triton X-100) for 10 min, blocked with PBS containing 1% BSA for 1 h, and sequentially incubated with a mouse anti-Flag primary antibody (1: 500, Sigma-Aldrich) and a FITC-conjugated anti-mouse secondary antibody (1:200, Jackson ImmunoResearch, West Grove, PA, USA) in PBS containing 1% BSA. Stained cells were mounted with SlowFade antifade medium (Invitrogen) and imaged with a LSM 800 laser-scanning confocal microscope (Carl Zeiss, Jena, Germany) using a Plan Apo 63 × 1.4 NA oil objective. The number of intracellular Myc-positive puncta was measured in cells with similar fluorescence intensities of Flag staining.

### Immunostaining of larval NMJs

Wandering third-instar larvae were dissected in Ca^2+^-free HL3 solution and fixed in PBS containing 4% formaldehyde for 20 min. Fixed larval fillets were washed with PBT-0.1 (PBS, 0.1% Triton X-100) and blocked with PBT-0.1 containing 0.2% BSA for 1 h. Samples were sequentially incubated with primary antibodies overnight at 4 °C and fluorescently-labeled secondary antibodies for 1 h at room temperature. The following monoclonal antibodies from the Developmental Studies Hybridoma Bank (DSHB, Iowa City, IA, USA) were used as primary antibodies: anti-HRP (1:200), anti-Futsch (1:50), anti-CSP (1:300), anti-NC82 (1:50), and anti-Dlg (1:500). Additional primary antibodies used were anti-P-Mad/PS1 (1:500) [39], anti-GluRIIC (1:200) [40], anti-P-Mad (1:100, Cell Signaling), and anti-Myc (1:200, Cell Signaling). FITC- and Cy3-conjugated secondary antibodies (Jackson ImmunoResearch) were used at 1:200. Images were captured with an LSM 800 laser-scanning confocal microscope using a C Apo 40× W or Plan Apo 63 × 1.4 NA objective.

Quantification of bouton number and satellite bouton number was performed at NMJ 6/7 and NMJ 4 in abdominal segment 2, as previously described [20]. Bouton number was normalized to muscle surface area. Statistical analysis was performed using SigmaPlot (Systat Software, San Jose, CA, USA). Comparisons were made by one-way ANOVA analysis with a post-hoc Turkey test. For comparison of only two samples, an unpaired Student’s *t*-test was used. Data are presented as mean ± SEM.

### FM1–43FX uptake assay

FM1–43FX dye uptake experiments were performed as described previously [9, 41]. Briefly, wandering third-instar larvae were dissected in Ca^2+^-free HL3 saline and then incubated in HL3 saline with 90 mM KCl, 5 mM CaCl_2_, and 4 μM FM1–43FX (Molecular Probes, Eugene, OR, USA) for 1 min. FM1–43FX-loaded samples were vigorously washed with Ca^2+^-free HL3 saline for 10 min, and fixed in PBS containing 4% formaldehyde, and washed three times in PBS. Images were collected using a Plan Apo 40 × 0.90 NA water-immersion objective on FV300 laser-scanning confocal microscope (Olympus, Tokyo, Japan).

### Histology, immunostaining, and TUNEL staining of adult brains

Heads from adult flies at 2, 10, 20, 30, and 40 days post-eclosion were fixed overnight in PBS containing 4% paraformaldehyde at 4 °C, embedded in paraffin, and subjected to serial 5-μm sectioning in a frontal orientation. Serial sections covering the entire brain were placed on a single slide and stained with hematoxylin and eosin (H&E) using a standard protocol. Vacuoles larger than 5 μm were counted throughout the entire brain.

For immunostaining analysis, brains from 20-day-old flies were dissected in ice-cold PBS, and fixed overnight in PBS containing 4% formaldehyde at 4 °C. Fixed brains were subsequently permeabilized in PBT-0.3 (PBS, 0.3% Triton X-100) for 1 h and blocked with PBT-0.3 containing 5% BSA for 1 h. The brains were sequentially incubated with primary antibodies for 48 h at 4 °C and fluorescently-labeled secondary antibodies for 24 h at 4 °C. The following primary antibodies were used in this study: anti-Elav (7E8A10, DSHB) at 1:10, anti-Repo (8D12, DSHB) at 1:10, and anti-cleaved caspase-3 (Cell Signaling) at 1:100. Antibody-stained brains were mounted in SlowFade antifade medium (Invitrogen). Fluorescent images were acquired with a LSM 800 laser-scanning confocal microscope using a C Apo 40× W objective.

TUNEL assays on paraffin sections of adult brains were performed using the In Situ Cell Death Detection Kit (Roche, Mannheim, Germany). Briefly, paraffin sections were dewaxed according to standard procedures. After washed with PBS, the sections were permeabilized in PBS containing 0.1% sodium citrate and 0.1% Triton X-100 for 15 min at room temperature. After washing with PBS, the samples were incubated with the TUNEL reaction mixture in a dark humid chamber for 1 h at 37 °C, prior to DAPI staining for 5 min at room temperature. TUNEL- and DAPI-positive cells were counted in three consecutive, middle frontal sections of adult brains.

### Adult climbing test

Adult locomotor ability was assayed as described previously [9]. For each genotype tested, approximately 100 flies were collected within 1 day of eclosion; aged for 2, 10, 20, 30, and 40 days; and placed into a glass graduated cylinder. After 5 min of adaptation to their environment, flies were gently vortexed for 5 s. The distance climbed by individual flies in a 30 s period was measured. Climbing assays were repeated 3 times for each genotype, and the results were averaged.

## Conclusions

In summary, our findings establish a novel role for the Gef26-Rap1 pathway in regulating BMP-dependent synaptic growth and neuronal survival. Regulation of surface expression of BMP receptors via endocytosis may represent an important underlying mechanism.

## Additional files

Additional file 1: Table S1. Quantification of NMJ parameters for the experiments in Fig. 1b. (PDF 1461 kb)

Additional file 2: Figure S1. Presynaptic requirement for Gef26 in synaptic growth regulation and characterization of satellite boutons. **a** Confocal images of anti-HRP-labeled NMJ 6/7 in *C155-GAL4*/+, *C155-GAL4*/+; *UAS-gef26* ^*RNAi*^/+, *BG57-GAL4*/+, and *BG57-GAL4*/*UAS-gef26* ^*RNAi*^ third-instar larvae*.* Scale bar, 20 μm. **b** Quantification of total bouton number and satellite bouton number*.* **c-e** Confocal images of NMJ 6/7 stained with anti-HRP and anti-NC82 (**c**), anti-CSP (**d**), or anti-Dlg (**e**) for wild-type and *gef26* ^*6*^ */Df* third-instar larvae. **f** Confocal images of NMJ 6/7 stained with anti-NC82 and anti-GluRIIC in wild-type and *gef26* ^*6*^ */Df* third-instar larvae. The number of NMJs analyzed is indicated in each bar. Data are expressed as mean ± SEM. *P < 0.001. (TIFF 20874 kb)

Additional file 3: Table S2. Quantification of NMJ parameters for the experiments in Additional file 2: Figure S1B. (PDF 247 kb)

Additional file 4: Table S3. Quantification of NMJ parameters for the experiments in Fig. 2b. (PDF 251 kb)

Additional file 5: Figure S2. *rap1* is required presynaptically for normal synaptic growth. **a** Confocal images of anti-HRP-labeled NMJ 6/7 in *C155-GAL4*/+, *C155-GAL4*/+; *UAS-rap1* ^*RNAi1*^/+, *C155-GAL4*/+; *UAS-rap1* ^*RNAi2*^/+, *BG57-GAL4*/+, *BG57-GAL4*/*UAS-rap1* ^*RNAi1*^, and *BG57-GAL4*/*UAS-rap1* ^*RNAi2*^ third-instar larvae. Scale bar, 20 μm. **b** Quantification of total bouton number and satellite bouton number. The number of NMJs analyzed is indicated in each bar. Data are expressed as mean ± SEM. *P < 0.001. (TIFF 21149 kb)

Additional file 6: Table S4. Quantification of NMJ parameters for the experiments in Additional file 5: Figure S2B. (PDF 247 kb)

Additional file 7: Table S5. Quantification of NMJ parameters for the experiments in Fig. 3. (PDF 252 kb)

Additional file 8: Table S6. Quantification of NMJ parameters for the experiments in Fig. 4. (PDF 253 kb)

Additional file 9: Table S7. Quantification of NMJ parameters for the experiments in Fig. 5b. (PDF 251 kb)

Additional file 10: Figure S3. *gef26* mutant NMJs show normal FM1–43FX dye uptake after nerve stimulation. **a** Confocal images of NMJ 6/7 boutons in wild-type and *gef26* ^*6*^/*Df* third instar larvae. NMJ synapses were stimulated for 1 min with 90 mM K^+^ and 5 mM Ca^2+^ in the presence of FM1–43FX. Scale bar, 20 μm. **b** Quantification of FM1–43FX fluorescence intensity. (TIFF 19338 kb)

Additional file 11: Figure S4. Progressive apoptotic cell death in *gef26* knockdown brains and reduced locomotor activities of flies transheterozygous for *gef26* and *rap1* or *dad*. **a** and **b** Neuron-specific knockdown of *gef26* expression causes age-dependent apoptotic cell death in the adult brain. **a** Confocal slices of 20-day-old *C155-GAL4*/+ and *C155-GAL4*/+; *UAS-gef26* ^*RNAi*^/+ brains labeled with TUNEL and DAPI. Scale bars, 20 μm. **b** Quantification of TUNEL-positive cells in three consecutive, middle frontal sections (5 μm thick) of *C155-GAL4*/+ and *C155-GAL4*/+; *UAS-gef26* ^*RNAi*^/+ brains. *n* = 4. **c** and **d** Reduced locomotor activities of flies transheterozygous for *gef26* and *rap1* or *dad*. **c** Distribution of the distance climbed by 30-day-old flies of the indicated genotypes over a 30 s period. **d** Quantification of average climbing distance for the genotypes indicated. All comparisons are with the *C155-GAL4*/+ control (**b**) or wild type (**d**): *P < 0.001. (TIFF 21040 kb)

## Abbreviations

BMP: Bone morphogenetic protein
CSP: Cysteine-string protein
*dfmr1*: *Drosophila* fragile X mental retardation 1
Dlg: Discs-large
Gbb: Glass bottom boat
GEF: Guanine nucleotide exchange factor
NMJ: Neuromuscular junction
P-Mad: Phosphorylated Mad
Tkv: Thickveins
