# Supplementary material for: The Rap activator Gef26 regulates synaptic growth and neuronal survival via inhibition of BMP signaling
Source: Mol Brain. 2017 Dec 28;10:62. doi: 10.1186/s13041-017-0342-7 (PMC5745669; doi:10.1186/s13041-017-0342-7)
Supplement: Supplementary file 1 — Quantification of NMJ parameters for the experiments in Fig. 1b. (PDF 1461 kb) [file 13041_2017_342_MOESM1_ESM.pdf]

Table S1. Quantification of NMJ parameters for experiments in Fig. 1B.

	Number of samples	Bouton number	p value vs WT	Muscle area (μm^2)X10^{-3}	p value vs WT	Bouton number /Muscle area (#/μm^2)X10^3	p value vs WT	Satellite bouton number	p value vs WT
**NMJ6/7**									
w^{1118} (WT)	20	118.90±1.81		88.67±1.48		1.35±0.03		14.10±0.67	
gef26^{6} /*gef26*^{6}	14	148.07±2.16	<0.001	86.20±1.77	1.731	1.72±0.03	<0.001	20.14±0.80	<0.001
gef26^{6} /*Df*	21	145.05±1.77	<0.001	85.88±1.45	1.349	1.69±0.03	<0.001	18.19±0.65	0.001
C155-GAL4 /+; *gef26*^{6} /*Df* ; *UAS-gef26* /+	21	116.24±1.77	<0.001	89.14±1.45	0.895	1.30±0.03	0.901	12.90±0.65	0.774
gef26^{6} /*Df* ; *BG57-GAL4* /*UAS-gef26*	17	144.41±1.96	<0.001	87.30±1.61	0.721	1.66±0.03	<0.001	19.41±0.72	<0.001
**NMJ4**									
w^{1118} (WT)	11	15.82±0.66		35.47±1.02		0.45±0.01		2.55±0.41	
gef26^{6} /*Df*	10	23.10±0.69	<0.001	33.94±1.07	<0.001	0.68±0.01	<0.001	8.10±0.43	<0.001
