# Supplementary figures and images for: The Rap activator Gef26 regulates synaptic growth and neuronal survival via inhibition of BMP signaling
Source: Mol Brain. 2017 Dec 28;10:62. doi: 10.1186/s13041-017-0342-7 (PMC5745669; doi:10.1186/s13041-017-0342-7)

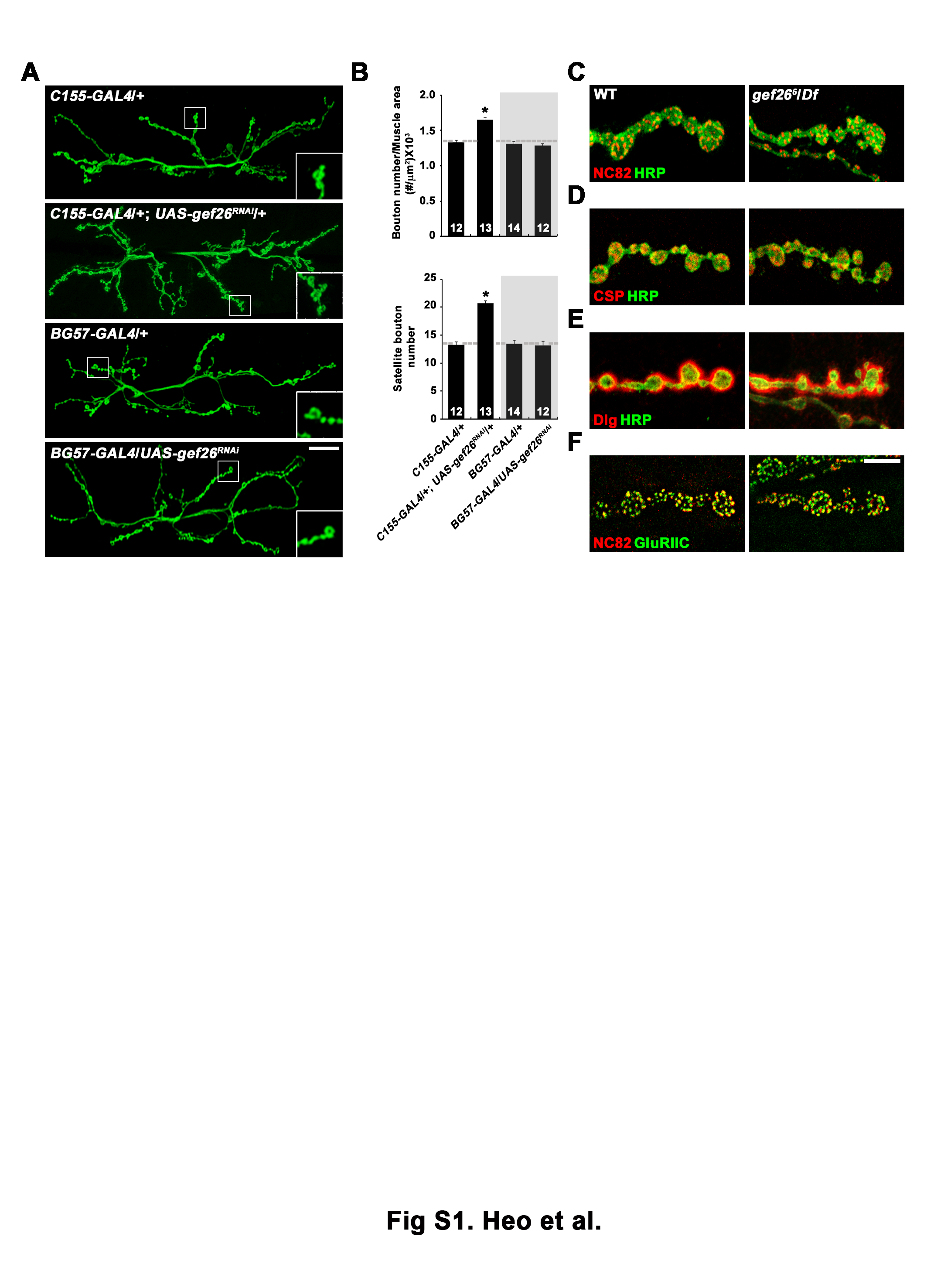

Supplement: Supplementary file 2 — Presynaptic requirement for Gef26 in synaptic growth regulation and characterization of satellite boutons. a Confocal images of anti-HRP-labeled NMJ 6/7 in C155-GAL4/+, C155-GAL4/+; UAS-gef26 RNAi/+, BG57-GAL4/+, and BG57-GAL4/UAS-gef26 RNAi third-instar larvae. Scale bar, 20 μm. b Quantification of total bouton number and satellite bouton number. c-e Confocal images of NMJ 6/7 stained with anti-HRP and anti-NC82 (c), anti-CSP (d), or anti-Dlg (e) for wild-type and gef26 6 /Df third-instar larvae. f Confocal images of NMJ 6/7 stained with anti-NC82 and anti-GluRIIC in wild-type and gef26 6 /Df third-instar larvae. The number of NMJs analyzed is indicated in each bar. Data are expressed as mean ± SEM. *P < 0.001. (TIFF 20874 kb) [file 13041_2017_342_MOESM2_ESM.tif]

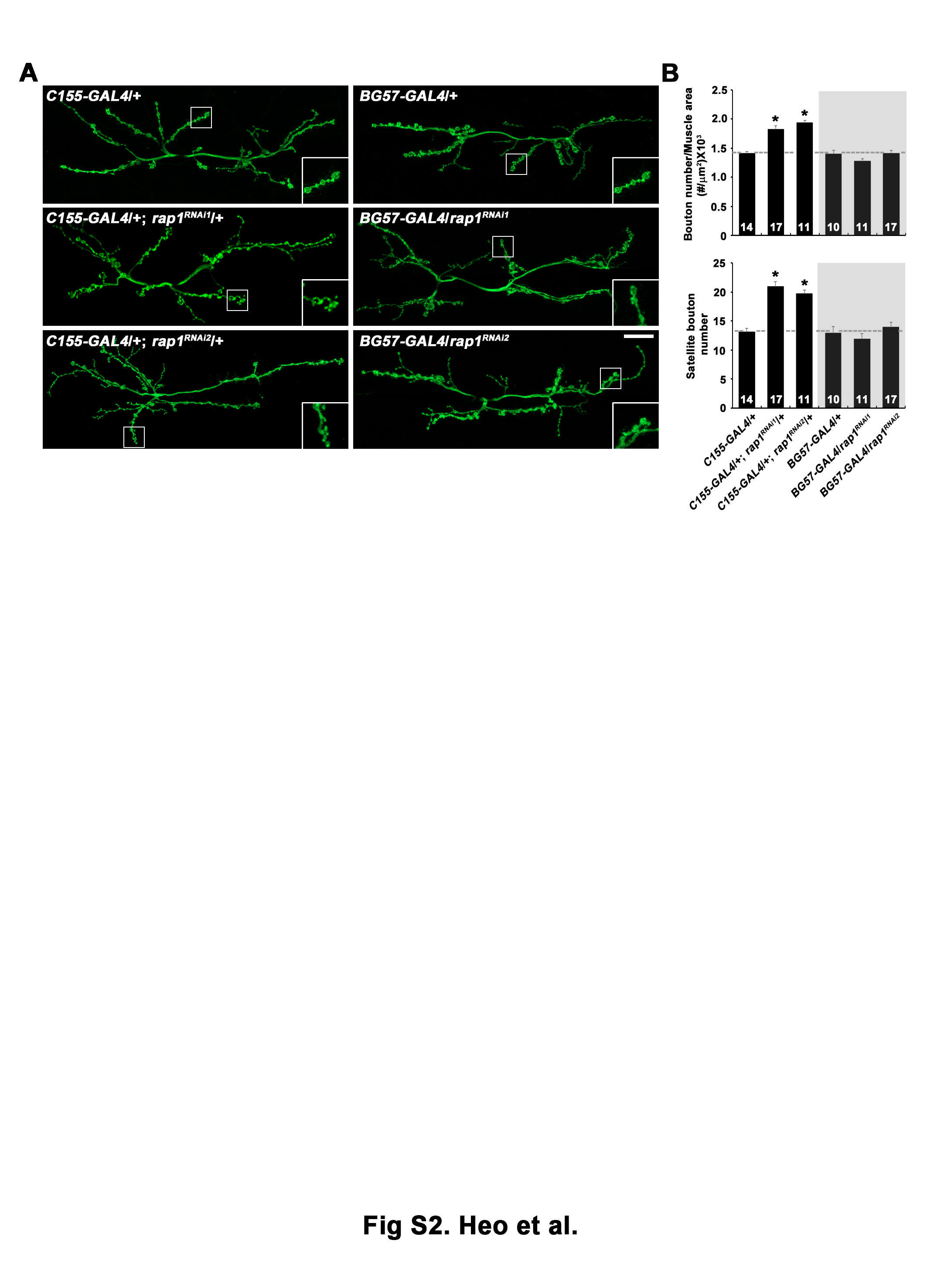

Supplement: Supplementary file 5 — rap1 is required presynaptically for normal synaptic growth. a Confocal images of anti-HRP-labeled NMJ 6/7 in C155-GAL4/+, C155-GAL4/+; UAS-rap1 RNAi1/+, C155-GAL4/+; UAS-rap1 RNAi2/+, BG57-GAL4/+, BG57-GAL4/UAS-rap1 RNAi1, and BG57-GAL4/UAS-rap1 RNAi2 third-instar larvae. Scale bar, 20 μm. b Quantification of total bouton number and satellite bouton number. The number of NMJs analyzed is indicated in each bar. Data are expressed as mean ± SEM. *P < 0.001. (TIFF 21149 kb) [file 13041_2017_342_MOESM5_ESM.tif]

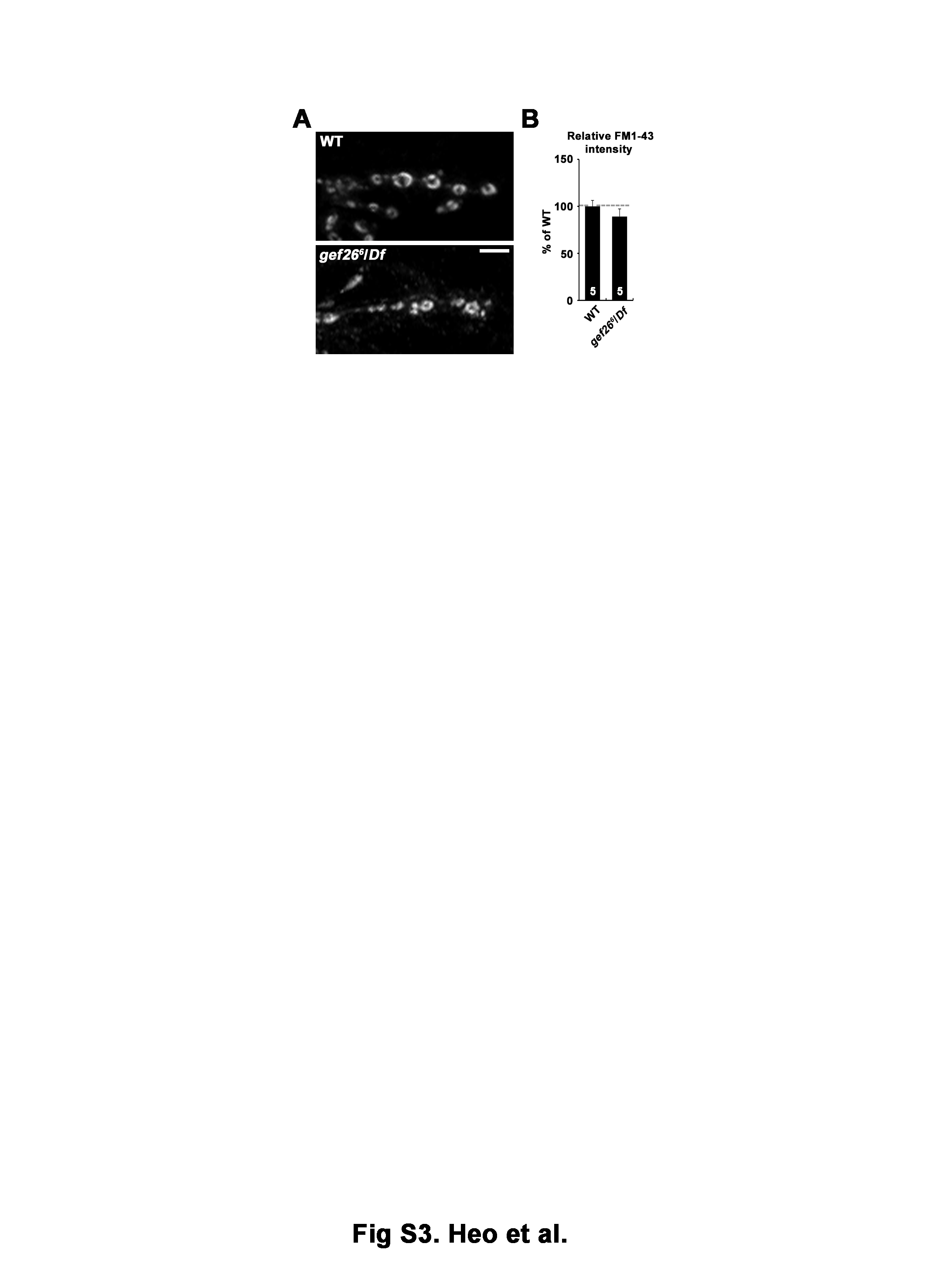

Supplement: Supplementary file 10 — gef26 mutant NMJs show normal FM1–43FX dye uptake after nerve stimulation. a Confocal images of NMJ 6/7 boutons in wild-type and gef26 6/Df third instar larvae. NMJ synapses were stimulated for 1 min with 90 mM K+ and 5 mM Ca2+ in the presence of FM1–43FX. Scale bar, 20 μm. b Quantification of FM1–43FX fluorescence intensity. (TIFF 19338 kb) [file 13041_2017_342_MOESM10_ESM.tif]

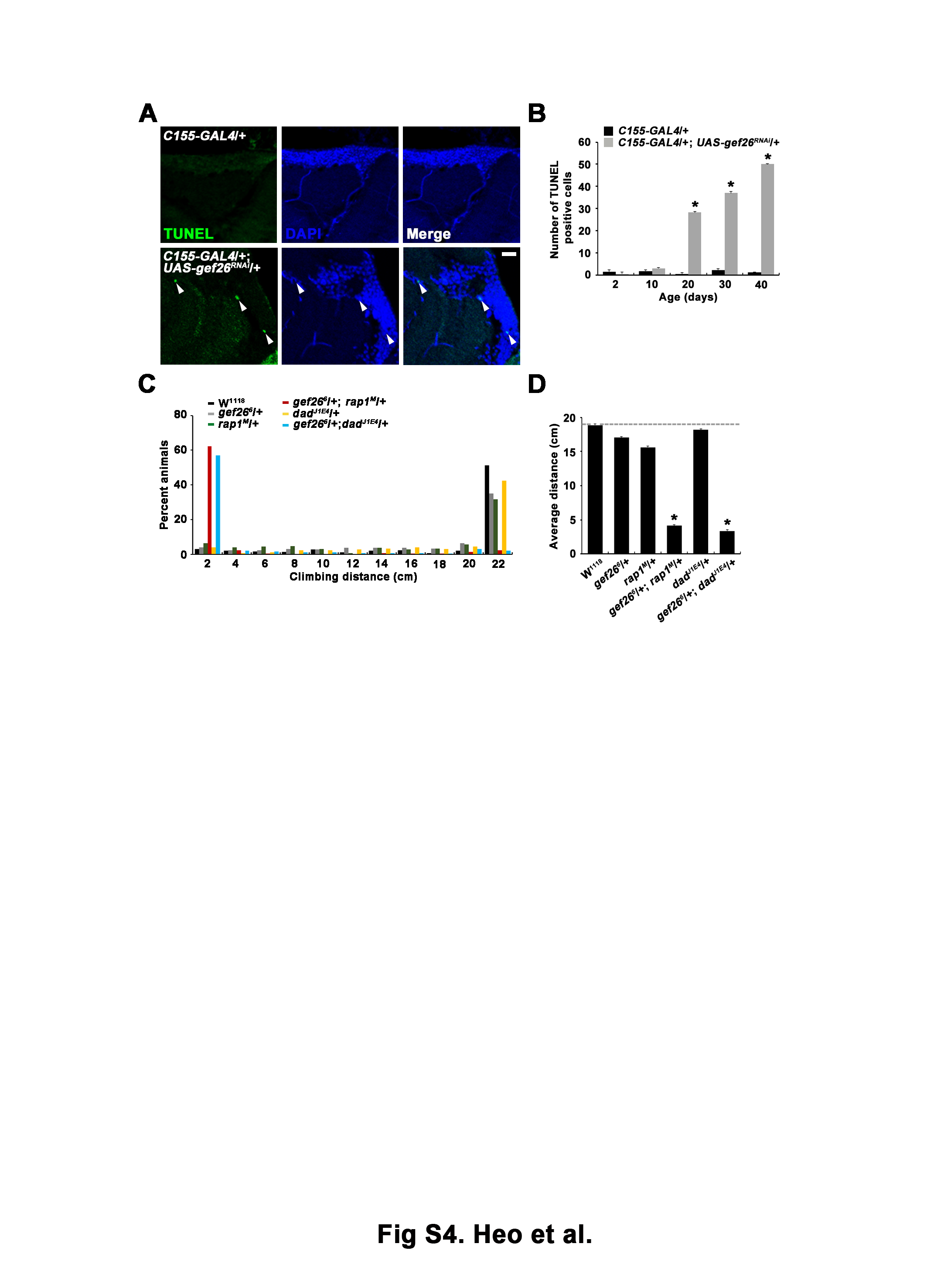

Supplement: Supplementary file 11 — Progressive apoptotic cell death in gef26 knockdown brains and reduced locomotor activities of flies transheterozygous for gef26 and rap1 or dad. a and b Neuron-specific knockdown of gef26 expression causes age-dependent apoptotic cell death in the adult brain. a Confocal slices of 20-day-old C155-GAL4/+ and C155-GAL4/+; UAS-gef26 RNAi/+ brains labeled with TUNEL and DAPI. Scale bars, 20 μm. b Quantification of TUNEL-positive cells in three consecutive, middle frontal sections (5 μm thick) of C155-GAL4/+ and C155-GAL4/+; UAS-gef26 RNAi/+ brains. n = 4. c and d Reduced locomotor activities of flies transheterozygous for gef26 and rap1 or dad. c Distribution of the distance climbed by 30-day-old flies of the indicated genotypes over a 30 s period. d Quantification of average climbing distance for the genotypes indicated. All comparisons are with the C155-GAL4/+ control (b) or wild type (d): *P < 0.001. (TIFF 21040 kb) [file 13041_2017_342_MOESM11_ESM.tif]
