# Supplementary material for: The Rap activator Gef26 regulates synaptic growth and neuronal survival via inhibition of BMP signaling
Source: Mol Brain. 2017 Dec 28;10:62. doi: 10.1186/s13041-017-0342-7 (PMC5745669; doi:10.1186/s13041-017-0342-7)
Supplement: Supplementary file 3 — Quantification of NMJ parameters for the experiments in Additional file 2: Figure S1B. (PDF 247 kb) [file 13041_2017_342_MOESM3_ESM.pdf]

Table S2. Quantification of NMJ parameters for experiments in Fig. S1B.

	**Number of samples**	**Bouton number**	p value vs *C155-GAL4* /+	**Muscle area (μm^2)X10^{-3}**	p value vs *C155-GAL4* /+	**Bouton number /Muscle area (#/μm^2)X10^3**	p value vs *C155-GAL4* /+	**Satellite bouton number**	p value vs *C155-GAL4* /+
C155-GAL4 /+	12	120.67±2.48		90.79±1.59		1.33±0.03		13.25±0.52	
C155-GAL4 /+; *UAS-gef26* ^{RNAi} /+	13	149.62±2.38	<0.001	90.47±1.53	0.851	1.65±0.03	<0.001	20.69±0.50	<0.001
			p value vs *BG57-GAL4* /+		p value vs *BG57-GAL4* /+		p value vs *BG57-GAL4* /+		p value vs *BG57-GAL4* /+
BG57-GAL4 /+	14	117.43±3.03		90.07±1.33		1.30±0.03		13.43±0.64	
BG57-GAL4 /*UAS-gef26* ^{RNAi}	12	116.92±2.52	0.901	91.30±1.49	0.561	1.28±0.03	0.654	13.17±0.70	0.794
