# Supplementary material for: The Rap activator Gef26 regulates synaptic growth and neuronal survival via inhibition of BMP signaling
Source: Mol Brain. 2017 Dec 28;10:62. doi: 10.1186/s13041-017-0342-7 (PMC5745669; doi:10.1186/s13041-017-0342-7)
Supplement: Supplementary file 4 — Quantification of NMJ parameters for the experiments in Fig. 2b. (PDF 251 kb) [file 13041_2017_342_MOESM4_ESM.pdf]

Table S3. Quantification of NMJ parameters for experiments in Fig. 2B.

	**Number of samples**	**Bouton number**	p value vs WT	**Muscle area (μm^2)X10^{-3}**	p value vs WT	**Bouton number /Muscle area (#/μm^2)X10^3**	p value vs WT	**Satellite bouton number**	p value vs WT
w^{1118} (WT)	10	127.67±3.55		92.93±0.93		1.37±0.03		13.78±0.72	
$rap1^{M}$/$rap1^{M}$	18	157.67±2.51	<0.001	90.44±0.66	0.857	1.74±0.02	<0.001	20.67±0.51	<0.001
$gef26^{6}$/+	12	130.83±3.21	1	91.50+0.84	0.997	1.43±0.03	0.996	13.50±0.65	1
$rap1^{M}$/+	12	133.00±3.21	0.993	90.38+0.84	0.899	1.48±0.03	0.873	13.50±0.65	1
$gef26^{6}$/+; *$rap1^{M}$*/+	14	170.40±5.67	<0.001	90.15±1.01	0.796	1.89±0.07	<0.001	21.07±1.09	<0.001
C155-GAL4/+	10	128.40±3.42	1	92.78±0.64	1	1.38±0.03	1	13.80±0.90	1
C155-GAL4/+; *UAS-Myc-$rap1^{CA}$*/+	15	96.33±2.79	<0.001	91.06±0.52	0.975	1.06±0.03	<0.001	8.53±0.74	0.003
$gef26^{6}$/Df	14	148.01±2.85	0.019	85.21±0.74	0.006	1.72±0.03	<0.001	20.14±0.58	<0.001
C155-GAL4/+; *$gef26^{6}$/Df*; *UAS-Myc-$rap1^{CA}$*/+	15	102.13±2.36	<0.001	90.37±0.50	0.859	1.13±0.02	0.017	9.53±0.26	0.036
