# Supplementary material for: The Rap activator Gef26 regulates synaptic growth and neuronal survival via inhibition of BMP signaling
Source: Mol Brain. 2017 Dec 28;10:62. doi: 10.1186/s13041-017-0342-7 (PMC5745669; doi:10.1186/s13041-017-0342-7)
Supplement: Supplementary file 6 — Quantification of NMJ parameters for the experiments in Additional file 5: Figure S2B. (PDF 247 kb) [file 13041_2017_342_MOESM6_ESM.pdf]

Table S4. Quantification of NMJ parameters for experiments in Fig. S2B.

	**Number of samples**	**Bouton number**	p value vs *C155-GAL4* /+	**Muscle area (μm^2)X10^{-3}**	p value vs *C155-GAL4* /+	**Bouton number /Muscle area (#/μm^2)X10^3**	p value vs *C155-GAL4* /+	**Satellite bouton number**	p value vs *C155-GAL4* /+
C155-GAL4 /+	14	128.93±2.58		91.83±1.31		1.41±0.03		13.14±0.57	
C155-GAL4 /+; *UAS-rap1* ^{RNAi1} /+	17	171.00±4.27	<0.001	94.03±1.28	0.753	1.82±0.05	<0.001	20.94±0.81	<0.001
C155-GAL4 /+; *UAS-rap1* ^{RNAi2} /+	11	184.36±2.90	<0.001	95.44±1.47	0.359	1.93±0.04	<0.001	19.73±0.65	<0.001
			p value vs *BG57-GAL4* /+		p value vs *BG57-GAL4* /+		p value vs *BG57-GAL4* /+		p value vs *BG57-GAL4* /+
BG57-GAL4 /+	10	124.00±5.71		88.99±1.04		1.39±0.07		12.89±1.13	
BG57-GAL4 /*UAS-rap1* ^{RNAi1}	11	107.82±5.14	0.249	84.64±1.84	0.507	1.27±0.04	0.920	11.91±0.90	0.920
BG57-GAL4 /*UAS-rap1* ^{RNAi2}	17	129.47±5.12	0.244	91.88±0.95	0.294	1.41±0.05	0.963	14.00±0.74	0.839
