# Supplementary material for: The Rap activator Gef26 regulates synaptic growth and neuronal survival via inhibition of BMP signaling
Source: Mol Brain. 2017 Dec 28;10:62. doi: 10.1186/s13041-017-0342-7 (PMC5745669; doi:10.1186/s13041-017-0342-7)
Supplement: Supplementary file 7 — Quantification of NMJ parameters for the experiments in Fig. 3. (PDF 252 kb) [file 13041_2017_342_MOESM7_ESM.pdf]

Table S5. Quantification of NMJ parameters for experiments in Fig. 3.

Fig. 3B

	**Number of samples**	**Bouton number**	p value vs WT	**Muscle area (μm^2)X10^{-3}**	p value vs WT	**Bouton number /Muscle area (#/μm^2)X10^3**	p value vs WT	**Satellite bouton number**	p value vs WT
w ^{1118} (WT)	11	117.45±2.09		89.90±0.55		1.31±0.02		13.18±0.70	
gef26 ^{6} */+*	11	116.54±2.30	1	90.73±0.35	0.999	1.28±0.02	0.999	12.09±0.71	0.956
rap1 ^{M} */+*	11	120.90±2.42	0.939	90.86±0.33	0.997	1.33±0.03	0.999	13.60±0.82	0.999
dad ^{J1E4} */+*	10	117.40±5.60	1	90.57±0.42	1	1.30±0.06	1	13.00±1.26	1
gef26 ^{6} */+; dad* ^{J1E4} */+*	11	147.18±2.92	0.868	90.24+2.44	1	1.64±0.05	<0.001	18.09±1.00	0.004
rap1 ^{M} *,+/+, dad* ^{J1E4}	12	162.00±3.70	<0.001	89.26+1.01	1	1.82±0.06	<0.001	22.83±0.99	<0.001

Fig. 3D

	**Number of samples**	**Bouton number**	p value vs WT	**Muscle area (μm^2)X10^{-3}**	p value vs WT	**Bouton number /Muscle area (#/μm^2)X10^3**	p value vs WT	**Satellite bouton number**	p value vs WT
w ^{1118} (WT)	12	120.75±2.53	1	87.05±3.46	1	1.40±0.08	1	14.33±1.14	1
gef26 ^{6} */Df*	10	146.20±4.77	<0.001	81.57±3.03	0.150	1.81±0.08	<0.001	18.80±1.27	0.014
tkv ^{7} */+*	10	125.30±1.96	0.990	89.78±0.80	0.887	1.39±0.01	1	12.70±0.40	0.901
tkv ^{7} *,gef26* ^{6} */+,Df*	15	110.60±2.93	<0.001	89.93±2.93	0.053	1.23±0.04	0.132	12.20±0.69	0.901
tkv ^{1} */tkv* ^{7}	13	88.69±3.87	<0.001	92.77±0.87	0.071	0.96±0.04	<0.001	6.69±0.65	<0.001
tkv ^{7} *,gef26* ^{6} */ tkv* ^{1} *,Df*	15	85.73±3.62	<0.001	92.79±0.60	<0.001	0.92±0.04	<0.001	4.67±0.53	<0.001
rap1 ^{M} */rap1* ^{M}	18	157.56±2.42	<0.001	90.44±2.29	0.223	1.74±0.05	<0.001	20.39±1.07	<0.001
tkv ^{7} */+; rap1* ^{M} */rap1* ^{M}	14	123.07±4.80	<0.001	92.02±0.33	<0.001	1.33±0.05	0.970	13.93±1.21	1
