# Supplementary material for: The Rap activator Gef26 regulates synaptic growth and neuronal survival via inhibition of BMP signaling
Source: Mol Brain. 2017 Dec 28;10:62. doi: 10.1186/s13041-017-0342-7 (PMC5745669; doi:10.1186/s13041-017-0342-7)
Supplement: Supplementary file 8 — Quantification of NMJ parameters for the experiments in Fig. 4. (PDF 253 kb) [file 13041_2017_342_MOESM8_ESM.pdf]

Table S6. Quantification of NMJ parameters for experiments in Fig. 4.

Fig. 4B

	**Number of samples**	**Bouton number**	p value vs WT	**Muscle area (μm^2)X10^{-3}**	p value vs WT	**Bouton number /Muscle area (#/μm^2)X10^3**	p value vs WT	**Satellite bouton number**	p value vs WT
w^{1118} (WT)	11	117.45±2.09		89.90±0.55		1.31±0.02		13.18±0.70	
$gef26^{6}$/+	11	116.73±2.37	1	90.72±0.35	0.985	1.29±0.03	0.998	12.55±0.68	0.996
$rap1^{M}$/+	11	120.36±2.05	0.979	90.86±0.33	0.967	1.32±0.02	0.999	12.81±0.67	1
$fmr1^{\Delta 50}$/+	14	121.36±1.92	0.908	90.50±0.38	0.996	1.34±0.02	0.968	12.64±0.68	0.997
$gef26^{6}$/+; $fmr1^{\Delta 50}$/+	11	149.91±4.71	<0.001	91.72±1.09	0.583	1.64±0.05	<0.001	19.82±1.29	<0.001
$rap1^{M}$,+/+,$fmr1^{\Delta 50}$	13	148.60±2.76	<0.001	90.50±0.31	0.997	1.64±0.03	<0.001	21.53±0.96	<0.001

Fig. 4G

	**Number of samples**	**Bouton number**	p value vs WT	**Muscle area (μm^2)X10^{-3}**	p value vs WT	**Bouton number /Muscle area (#/μm^2)X10^3**	p value vs WT	**Satellite bouton number**	p value vs WT
**Vinblastine (-)**									
w^{1118} (WT)	11	115.91±2.26		90.27±0.78		1.28±0.02		12.45±1.54	
$gef26^{6}$/Df	10	137.70±0.99	<0.001	90.02±0.53	1	1.53±0.01	<0.001	178.40±0.45	0.001
$rap1^{M}$/$rap1^{M}$	11	150.73±2.77	<0.001	89.75±0.33	0.998	1.68±0.03	<0.001	20.82±1.21	<0.001
**Vinblastine (+)**									
			p value vs WT (non-treated)		p value vs WT (non-treated)		p value vs WT (non-treated)		p value vs WT (non-treated)
w^{1118} (WT)	11	120.54±2.72	0.757	90.51±0.59	1	1.33±0.03	0.801	12.81±0.97	1
$gef26^{6}$/Df	15	122.86±2.19	0.221	90.12±0.37	1	1.36±0.02	0.234	12.93±0.66	1
$rap1^{M}$/$rap1^{M}$	14	124.57±2.61	0.066	90.64±0.51	1	1.37±0.03	0.133	14.50±1.05	0.793
