# Supplementary material for: The Rap activator Gef26 regulates synaptic growth and neuronal survival via inhibition of BMP signaling
Source: Mol Brain. 2017 Dec 28;10:62. doi: 10.1186/s13041-017-0342-7 (PMC5745669; doi:10.1186/s13041-017-0342-7)
Supplement: Supplementary file 9 — Quantification of NMJ parameters for the experiments in Fig. 5b. (PDF 251 kb) [file 13041_2017_342_MOESM9_ESM.pdf]

Table S7. Quantification of NMJ parameters for experiments in Fig. 5B.

	Number of samples	Bouton number	p value vs WT	Muscle area (μm^2)X10^{-3}	p value vs WT	Bouton number /Muscle area (#/μm^2)X10^3	p value vs WT	Satellite bouton number	p value vs WT
w ^{1118} (WT)	10	120.39±2.66		87.25±3.47		1.40±0.07		14.29±0.93	
gef26 ^{6}/+	10	118.30±2.98	0.996	91.05±2.09	n.s	1.30±0.03	0.545	14.50±0.65	1
endoA $^{\Delta 4}$/+	10	132.10±3.31	0.075	90.21±0.79	n.s	1.46±0.03	0.906	15.70±0.52	0.843
dap160 $^{\Delta 1}$/+	13	120.85±1.55	1	88.56±0.91	n.s	1.36±0.01	0.539	13.31±0.44	0.947
nwk ^{2}/+	9	124.22±5.05	0.995	87.56±1.00	n.s	1.42±0.05	1	16.89±2.19	0.663
gef26 ^{6}/+; *endoA* $^{\Delta 4}$/+	15	176.27±3.26	<0.001	89.03±0.67	n.s	1.98±0.04	<0.001	25.00±1.05	<0.001
gef26 ^{6}/*dap160* $^{\Delta 1}$	15	151.57±2.96	<0.001	88.95±0.85	n.s	1.71±0.04	<0.001	19.14±0.82	<0.001
gef26 ^{6}/+; *nwk* ^{2}/+	14	113.43±3.87	0.786	86.82±0.98	n.s	1.30±0.04	0.666	12.29±0.77	0.805
